# Supplementary material for: Transcriptional Comparison of Genes Associated with Photosynthesis, Photorespiration, and Photo-Assimilate Allocation and Metabolic Profiling of Rice Species
Source: Int J Mol Sci. 2022 Aug 10;23(16):8901. doi: 10.3390/ijms23168901 (PMC9408291; doi:10.3390/ijms23168901)
Supplement: Supplementary file 1 [file ijms-23-08901-s001.zip › ijms-1826694-supplementary.pptx]

## Slide 1
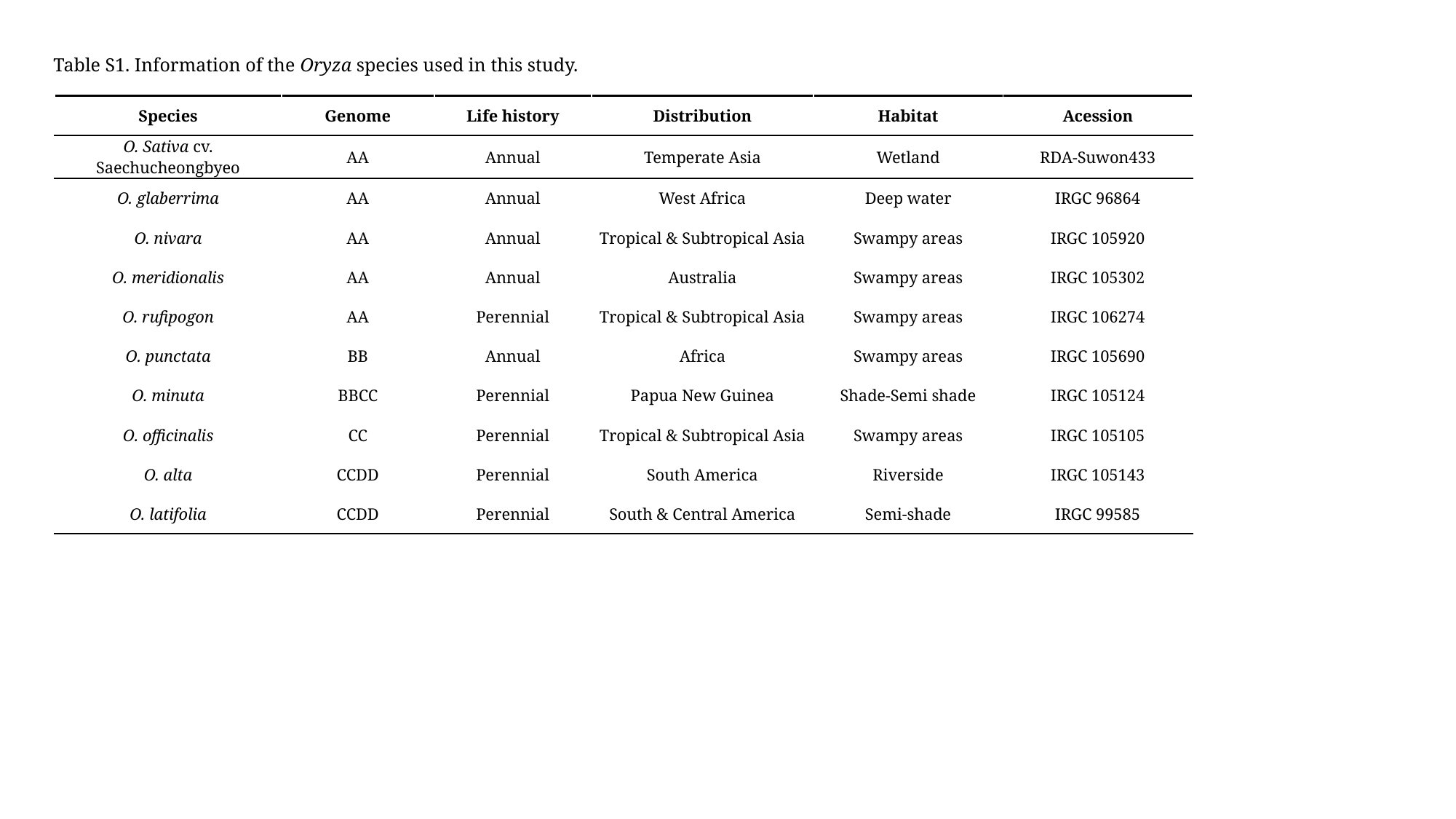

Table S1. Information of the Oryza species used in this study.
| Species | Genome | Life history | Distribution | Habitat | Acession |
| --- | --- | --- | --- | --- | --- |
| O. Sativa cv. Saechucheongbyeo | AA | Annual | Temperate Asia | Wetland | RDA-Suwon433 |
| O. glaberrima | AA | Annual | West Africa | Deep water | IRGC 96864 |
| O. nivara | AA | Annual | Tropical & Subtropical Asia | Swampy areas | IRGC 105920 |
| O. meridionalis | AA | Annual | Australia | Swampy areas | IRGC 105302 |
| O. rufipogon | AA | Perennial | Tropical & Subtropical Asia | Swampy areas | IRGC 106274 |
| O. punctata | BB | Annual | Africa | Swampy areas | IRGC 105690 |
| O. minuta | BBCC | Perennial | Papua New Guinea | Shade-Semi shade | IRGC 105124 |
| O. officinalis | CC | Perennial | Tropical & Subtropical Asia | Swampy areas | IRGC 105105 |
| O. alta | CCDD | Perennial | South America | Riverside | IRGC 105143 |
| O. latifolia | CCDD | Perennial | South & Central America | Semi-shade | IRGC 99585 |

## Slide 2
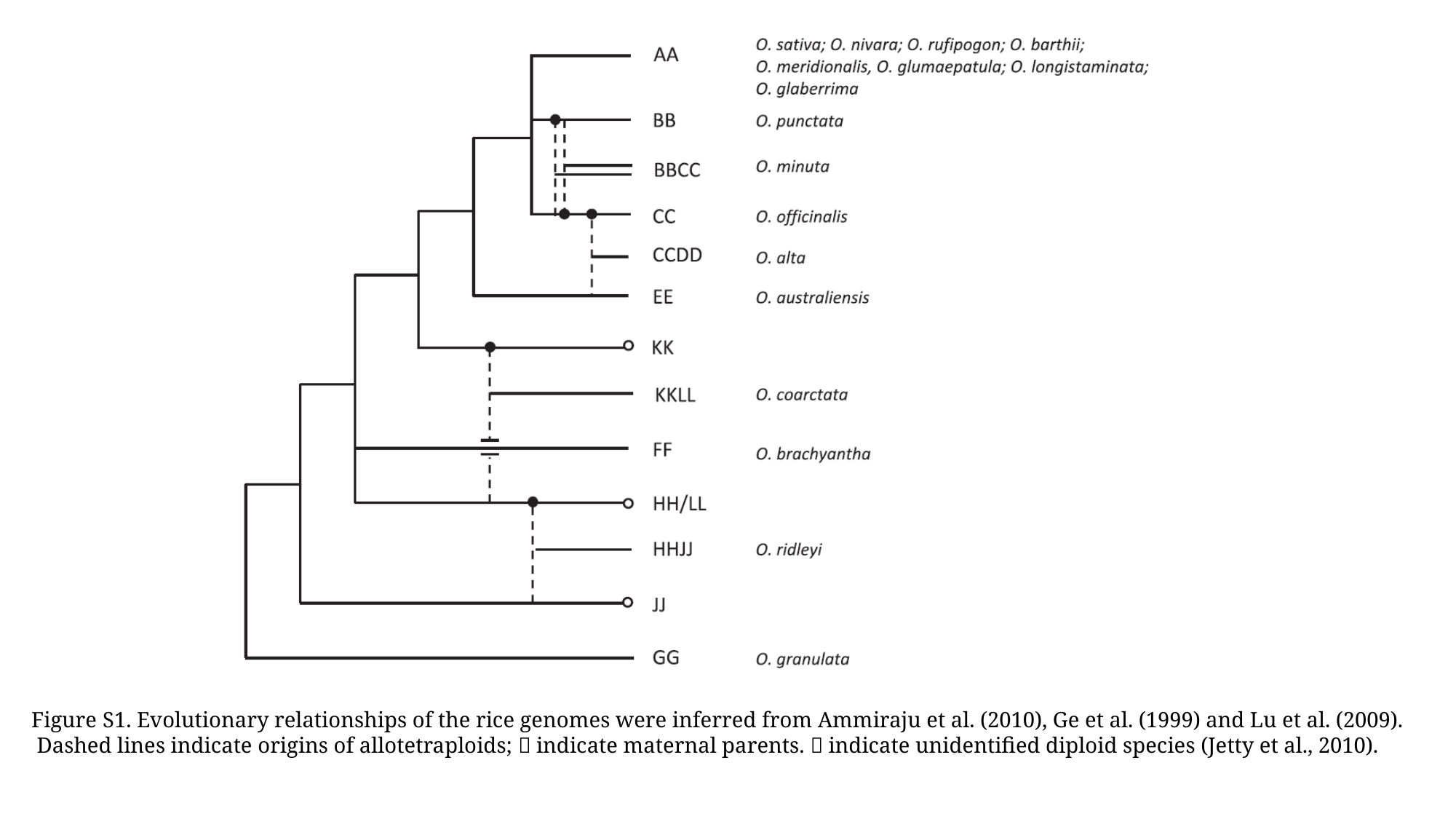

Figure S1. Evolutionary relationships of the rice genomes were inferred from Ammiraju et al. (2010), Ge et al. (1999) and Lu et al. (2009).
 Dashed lines indicate origins of allotetraploids;  indicate maternal parents.  indicate unidentified diploid species (Jetty et al., 2010).

## Slide 3
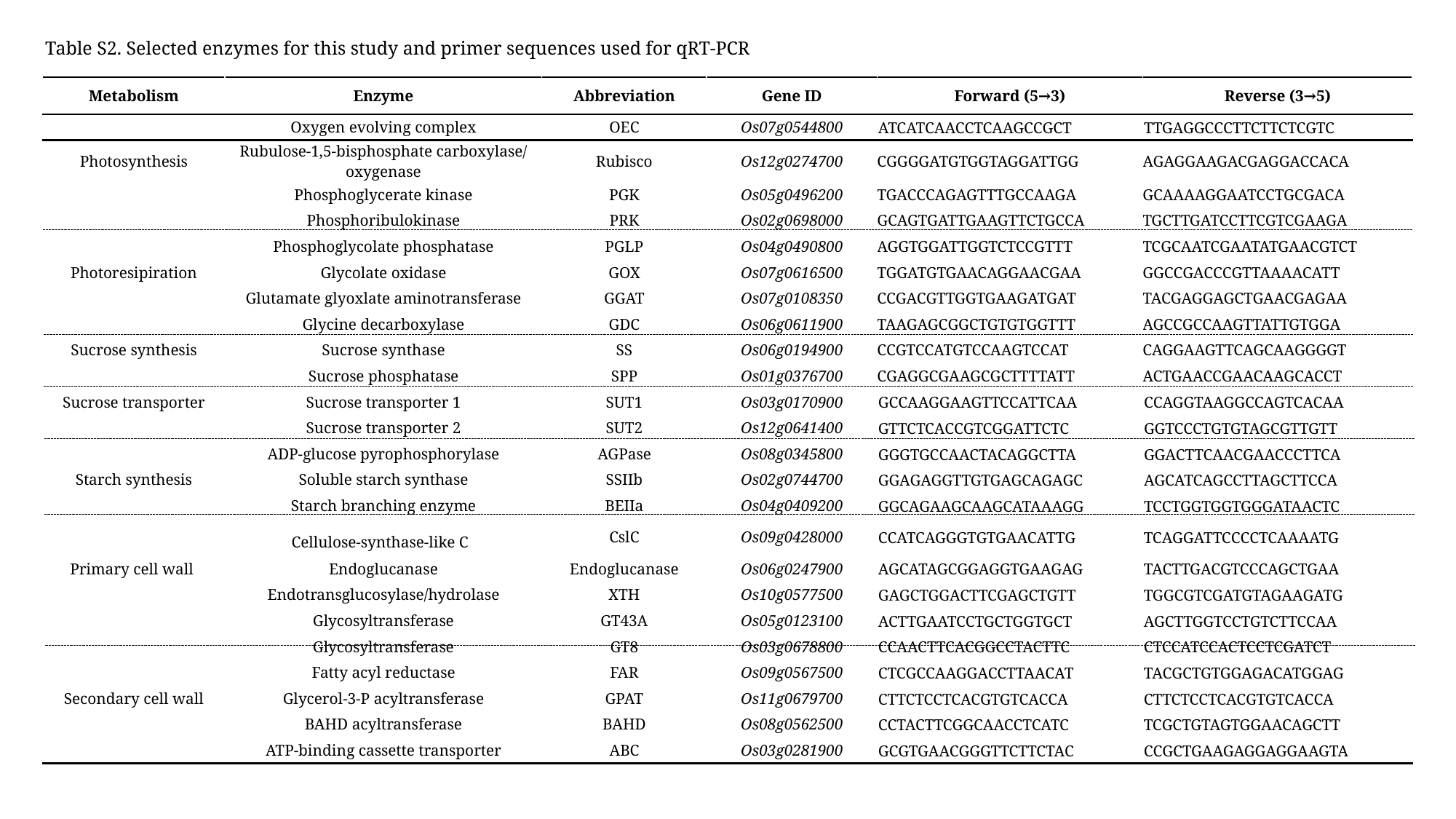

Table S2. Selected enzymes for this study and primer sequences used for qRT-PCR
| Metabolism | Enzyme | Abbreviation | Gene ID | Forward (5→3) | Reverse (3→5) |
| --- | --- | --- | --- | --- | --- |
| | Oxygen evolving complex | OEC | Os07g0544800 | ATCATCAACCTCAAGCCGCT | TTGAGGCCCTTCTTCTCGTC |
| Photosynthesis | Rubulose-1,5-bisphosphate carboxylase/oxygenase | Rubisco | Os12g0274700 | CGGGGATGTGGTAGGATTGG | AGAGGAAGACGAGGACCACA |
| | Phosphoglycerate kinase | PGK | Os05g0496200 | TGACCCAGAGTTTGCCAAGA | GCAAAAGGAATCCTGCGACA |
| | Phosphoribulokinase | PRK | Os02g0698000 | GCAGTGATTGAAGTTCTGCCA | TGCTTGATCCTTCGTCGAAGA |
| | Phosphoglycolate phosphatase | PGLP | Os04g0490800 | AGGTGGATTGGTCTCCGTTT | TCGCAATCGAATATGAACGTCT |
| Photoresipiration | Glycolate oxidase | GOX | Os07g0616500 | TGGATGTGAACAGGAACGAA | GGCCGACCCGTTAAAACATT |
| | Glutamate glyoxlate aminotransferase | GGAT | Os07g0108350 | CCGACGTTGGTGAAGATGAT | TACGAGGAGCTGAACGAGAA |
| | Glycine decarboxylase | GDC | Os06g0611900 | TAAGAGCGGCTGTGTGGTTT | AGCCGCCAAGTTATTGTGGA |
| Sucrose synthesis | Sucrose synthase | SS | Os06g0194900 | CCGTCCATGTCCAAGTCCAT | CAGGAAGTTCAGCAAGGGGT |
| | Sucrose phosphatase | SPP | Os01g0376700 | CGAGGCGAAGCGCTTTTATT | ACTGAACCGAACAAGCACCT |
| Sucrose transporter | Sucrose transporter 1 | SUT1 | Os03g0170900 | GCCAAGGAAGTTCCATTCAA | CCAGGTAAGGCCAGTCACAA |
| | Sucrose transporter 2 | SUT2 | Os12g0641400 | GTTCTCACCGTCGGATTCTC | GGTCCCTGTGTAGCGTTGTT |
| | ADP-glucose pyrophosphorylase | AGPase | Os08g0345800 | GGGTGCCAACTACAGGCTTA | GGACTTCAACGAACCCTTCA |
| Starch synthesis | Soluble starch synthase | SSIIb | Os02g0744700 | GGAGAGGTTGTGAGCAGAGC | AGCATCAGCCTTAGCTTCCA |
| | Starch branching enzyme | BEIIa | Os04g0409200 | GGCAGAAGCAAGCATAAAGG | TCCTGGTGGTGGGATAACTC |
| | Cellulose-synthase-like C | CslC | Os09g0428000 | CCATCAGGGTGTGAACATTG | TCAGGATTCCCCTCAAAATG |
| Primary cell wall | Endoglucanase | Endoglucanase | Os06g0247900 | AGCATAGCGGAGGTGAAGAG | TACTTGACGTCCCAGCTGAA |
| | Endotransglucosylase/hydrolase | XTH | Os10g0577500 | GAGCTGGACTTCGAGCTGTT | TGGCGTCGATGTAGAAGATG |
| | Glycosyltransferase | GT43A | Os05g0123100 | ACTTGAATCCTGCTGGTGCT | AGCTTGGTCCTGTCTTCCAA |
| | Glycosyltransferase | GT8 | Os03g0678800 | CCAACTTCACGGCCTACTTC | CTCCATCCACTCCTCGATCT |
| | Fatty acyl reductase | FAR | Os09g0567500 | CTCGCCAAGGACCTTAACAT | TACGCTGTGGAGACATGGAG |
| Secondary cell wall | Glycerol-3-P acyltransferase | GPAT | Os11g0679700 | CTTCTCCTCACGTGTCACCA | CTTCTCCTCACGTGTCACCA |
| | BAHD acyltransferase | BAHD | Os08g0562500 | CCTACTTCGGCAACCTCATC | TCGCTGTAGTGGAACAGCTT |
| | ATP-binding cassette transporter | ABC | Os03g0281900 | GCGTGAACGGGTTCTTCTAC | CCGCTGAAGAGGAGGAAGTA |

## Slide 4
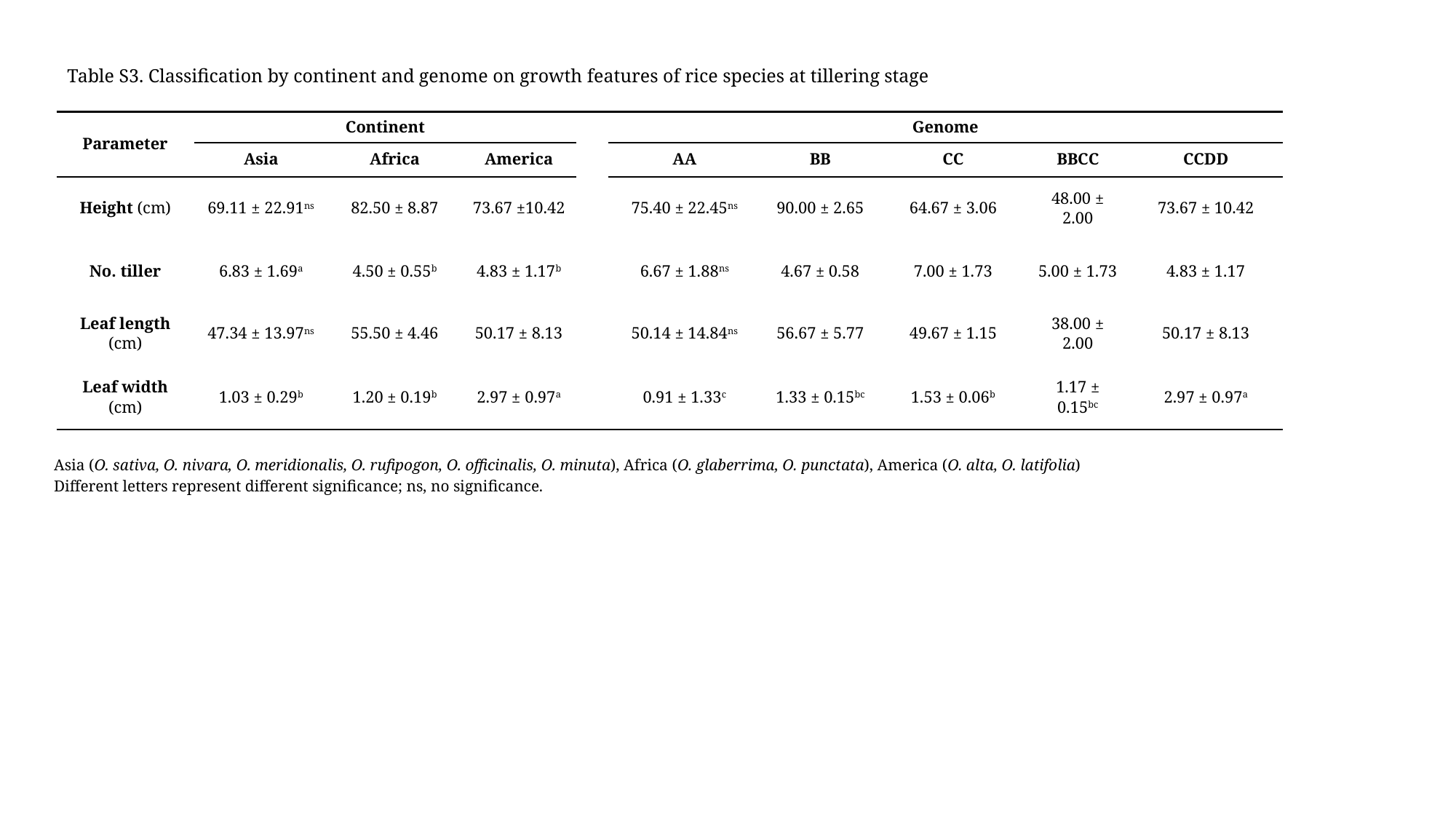

Table S3. Classification by continent and genome on growth features of rice species at tillering stage
| Parameter | Continent | | | | Genome | | | | |
| --- | --- | --- | --- | --- | --- | --- | --- | --- | --- |
| | Asia | Africa | America | | AA | BB | CC | BBCC | CCDD |
| Height (cm) | 69.11 ± 22.91ns | 82.50 ± 8.87 | 73.67 ±10.42 | | 75.40 ± 22.45ns | 90.00 ± 2.65 | 64.67 ± 3.06 | 48.00 ± 2.00 | 73.67 ± 10.42 |
| No. tiller | 6.83 ± 1.69a | 4.50 ± 0.55b | 4.83 ± 1.17b | | 6.67 ± 1.88ns | 4.67 ± 0.58 | 7.00 ± 1.73 | 5.00 ± 1.73 | 4.83 ± 1.17 |
| Leaf length (cm) | 47.34 ± 13.97ns | 55.50 ± 4.46 | 50.17 ± 8.13 | | 50.14 ± 14.84ns | 56.67 ± 5.77 | 49.67 ± 1.15 | 38.00 ± 2.00 | 50.17 ± 8.13 |
| Leaf width (cm) | 1.03 ± 0.29b | 1.20 ± 0.19b | 2.97 ± 0.97a | | 0.91 ± 1.33c | 1.33 ± 0.15bc | 1.53 ± 0.06b | 1.17 ± 0.15bc | 2.97 ± 0.97a |
Asia (O. sativa, O. nivara, O. meridionalis, O. rufipogon, O. officinalis, O. minuta), Africa (O. glaberrima, O. punctata), America (O. alta, O. latifolia)
Different letters represent different significance; ns, no significance.

## Slide 5
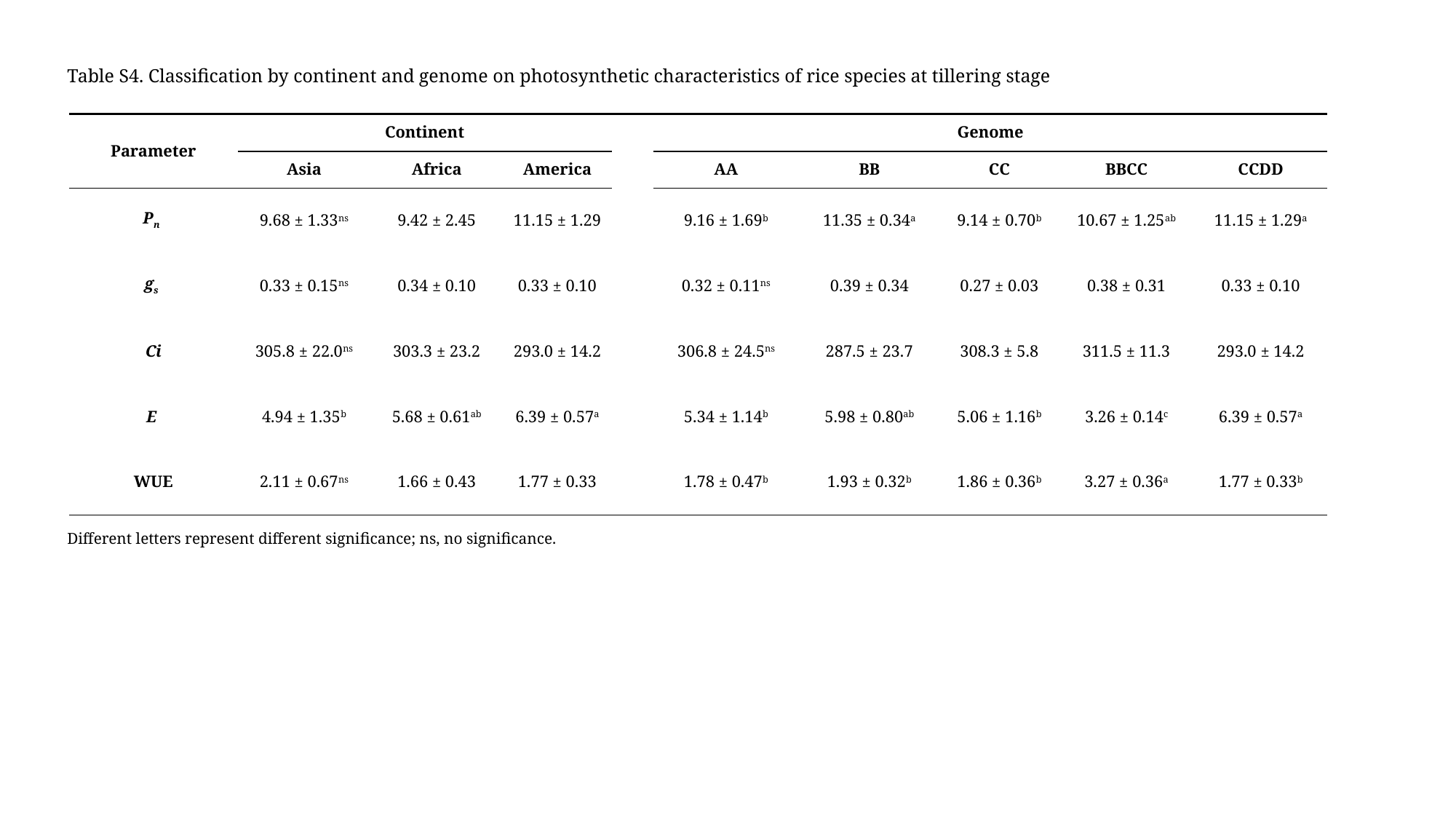

Table S4. Classification by continent and genome on photosynthetic characteristics of rice species at tillering stage
| Parameter | Continent | | | | Genome | | | | |
| --- | --- | --- | --- | --- | --- | --- | --- | --- | --- |
| | Asia | Africa | America | | AA | BB | CC | BBCC | CCDD |
| Pn | 9.68 ± 1.33ns | 9.42 ± 2.45 | 11.15 ± 1.29 | | 9.16 ± 1.69b | 11.35 ± 0.34a | 9.14 ± 0.70b | 10.67 ± 1.25ab | 11.15 ± 1.29a |
| gs | 0.33 ± 0.15ns | 0.34 ± 0.10 | 0.33 ± 0.10 | | 0.32 ± 0.11ns | 0.39 ± 0.34 | 0.27 ± 0.03 | 0.38 ± 0.31 | 0.33 ± 0.10 |
| Ci | 305.8 ± 22.0ns | 303.3 ± 23.2 | 293.0 ± 14.2 | | 306.8 ± 24.5ns | 287.5 ± 23.7 | 308.3 ± 5.8 | 311.5 ± 11.3 | 293.0 ± 14.2 |
| E | 4.94 ± 1.35b | 5.68 ± 0.61ab | 6.39 ± 0.57a | | 5.34 ± 1.14b | 5.98 ± 0.80ab | 5.06 ± 1.16b | 3.26 ± 0.14c | 6.39 ± 0.57a |
| WUE | 2.11 ± 0.67ns | 1.66 ± 0.43 | 1.77 ± 0.33 | | 1.78 ± 0.47b | 1.93 ± 0.32b | 1.86 ± 0.36b | 3.27 ± 0.36a | 1.77 ± 0.33b |
Different letters represent different significance; ns, no significance.

## Slide 6
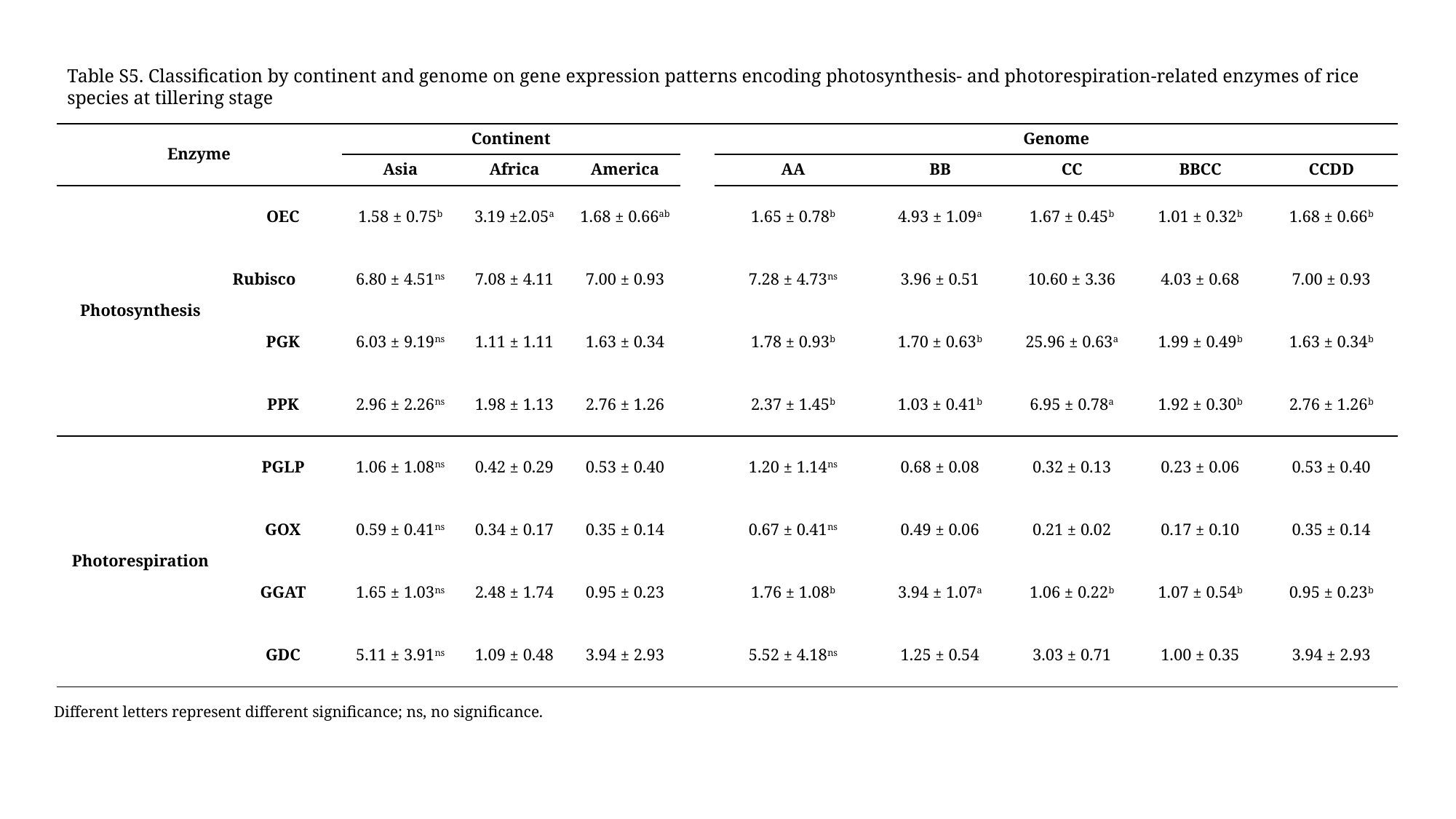

Table S5. Classification by continent and genome on gene expression patterns encoding photosynthesis- and photorespiration-related enzymes of rice species at tillering stage
| Enzyme | | Continent | | | | Genome | | | | |
| --- | --- | --- | --- | --- | --- | --- | --- | --- | --- | --- |
| | | Asia | Africa | America | | AA | BB | CC | BBCC | CCDD |
| Photosynthesis | OEC | 1.58 ± 0.75b | 3.19 ±2.05a | 1.68 ± 0.66ab | | 1.65 ± 0.78b | 4.93 ± 1.09a | 1.67 ± 0.45b | 1.01 ± 0.32b | 1.68 ± 0.66b |
| | Rubisco | 6.80 ± 4.51ns | 7.08 ± 4.11 | 7.00 ± 0.93 | | 7.28 ± 4.73ns | 3.96 ± 0.51 | 10.60 ± 3.36 | 4.03 ± 0.68 | 7.00 ± 0.93 |
| | PGK | 6.03 ± 9.19ns | 1.11 ± 1.11 | 1.63 ± 0.34 | | 1.78 ± 0.93b | 1.70 ± 0.63b | 25.96 ± 0.63a | 1.99 ± 0.49b | 1.63 ± 0.34b |
| | PPK | 2.96 ± 2.26ns | 1.98 ± 1.13 | 2.76 ± 1.26 | | 2.37 ± 1.45b | 1.03 ± 0.41b | 6.95 ± 0.78a | 1.92 ± 0.30b | 2.76 ± 1.26b |
| Photorespiration | PGLP | 1.06 ± 1.08ns | 0.42 ± 0.29 | 0.53 ± 0.40 | | 1.20 ± 1.14ns | 0.68 ± 0.08 | 0.32 ± 0.13 | 0.23 ± 0.06 | 0.53 ± 0.40 |
| | GOX | 0.59 ± 0.41ns | 0.34 ± 0.17 | 0.35 ± 0.14 | | 0.67 ± 0.41ns | 0.49 ± 0.06 | 0.21 ± 0.02 | 0.17 ± 0.10 | 0.35 ± 0.14 |
| | GGAT | 1.65 ± 1.03ns | 2.48 ± 1.74 | 0.95 ± 0.23 | | 1.76 ± 1.08b | 3.94 ± 1.07a | 1.06 ± 0.22b | 1.07 ± 0.54b | 0.95 ± 0.23b |
| | GDC | 5.11 ± 3.91ns | 1.09 ± 0.48 | 3.94 ± 2.93 | | 5.52 ± 4.18ns | 1.25 ± 0.54 | 3.03 ± 0.71 | 1.00 ± 0.35 | 3.94 ± 2.93 |
Different letters represent different significance; ns, no significance.

## Slide 7
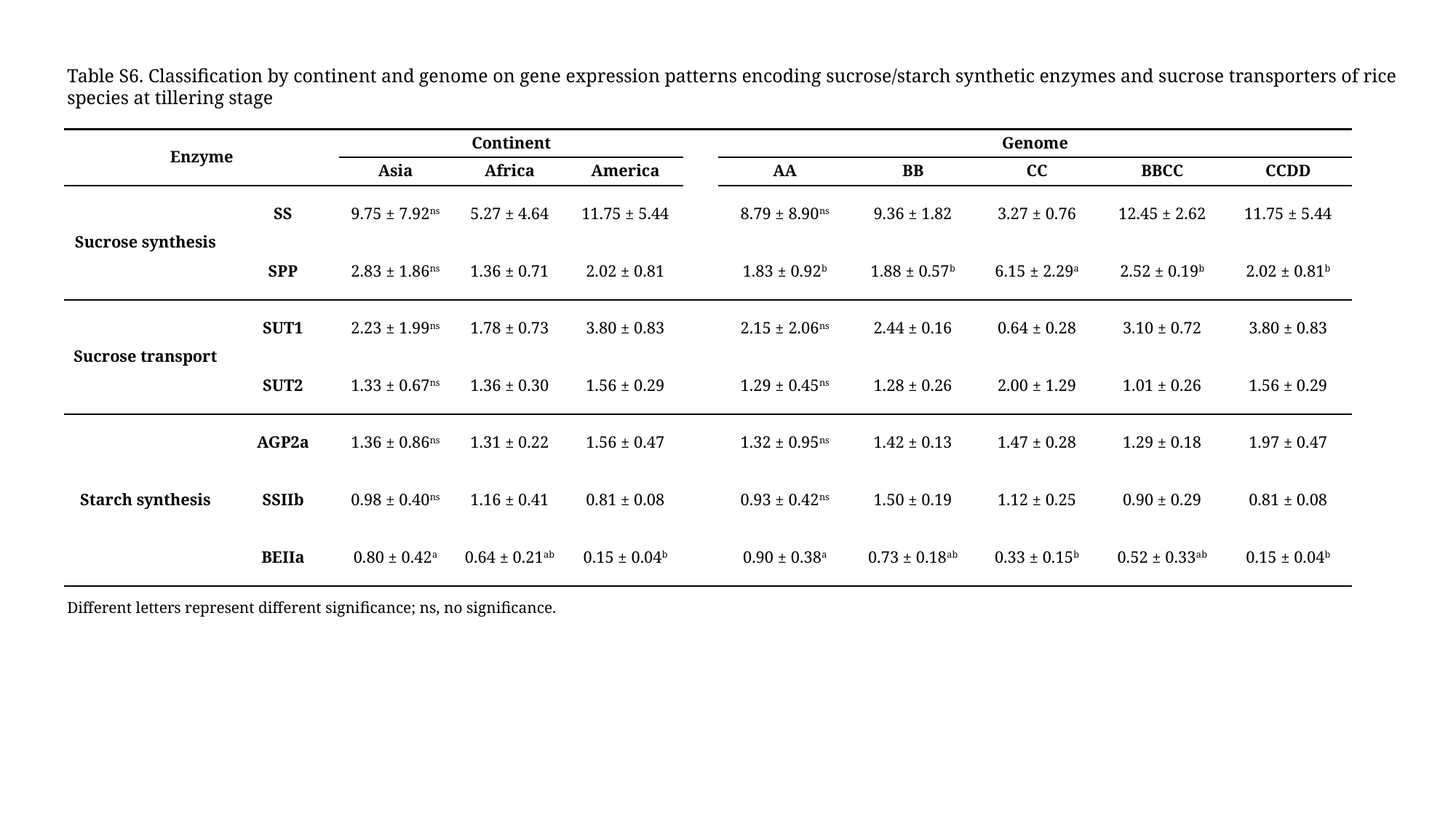

Table S6. Classification by continent and genome on gene expression patterns encoding sucrose/starch synthetic enzymes and sucrose transporters of rice species at tillering stage
| Enzyme | | Continent | | | | Genome | | | | |
| --- | --- | --- | --- | --- | --- | --- | --- | --- | --- | --- |
| | | Asia | Africa | America | | AA | BB | CC | BBCC | CCDD |
| Sucrose synthesis | SS | 9.75 ± 7.92ns | 5.27 ± 4.64 | 11.75 ± 5.44 | | 8.79 ± 8.90ns | 9.36 ± 1.82 | 3.27 ± 0.76 | 12.45 ± 2.62 | 11.75 ± 5.44 |
| | SPP | 2.83 ± 1.86ns | 1.36 ± 0.71 | 2.02 ± 0.81 | | 1.83 ± 0.92b | 1.88 ± 0.57b | 6.15 ± 2.29a | 2.52 ± 0.19b | 2.02 ± 0.81b |
| Sucrose transport | SUT1 | 2.23 ± 1.99ns | 1.78 ± 0.73 | 3.80 ± 0.83 | | 2.15 ± 2.06ns | 2.44 ± 0.16 | 0.64 ± 0.28 | 3.10 ± 0.72 | 3.80 ± 0.83 |
| | SUT2 | 1.33 ± 0.67ns | 1.36 ± 0.30 | 1.56 ± 0.29 | | 1.29 ± 0.45ns | 1.28 ± 0.26 | 2.00 ± 1.29 | 1.01 ± 0.26 | 1.56 ± 0.29 |
| Starch synthesis | AGP2a | 1.36 ± 0.86ns | 1.31 ± 0.22 | 1.56 ± 0.47 | | 1.32 ± 0.95ns | 1.42 ± 0.13 | 1.47 ± 0.28 | 1.29 ± 0.18 | 1.97 ± 0.47 |
| | SSIIb | 0.98 ± 0.40ns | 1.16 ± 0.41 | 0.81 ± 0.08 | | 0.93 ± 0.42ns | 1.50 ± 0.19 | 1.12 ± 0.25 | 0.90 ± 0.29 | 0.81 ± 0.08 |
| | BEIIa | 0.80 ± 0.42a | 0.64 ± 0.21ab | 0.15 ± 0.04b | | 0.90 ± 0.38a | 0.73 ± 0.18ab | 0.33 ± 0.15b | 0.52 ± 0.33ab | 0.15 ± 0.04b |
Different letters represent different significance; ns, no significance.

## Slide 8
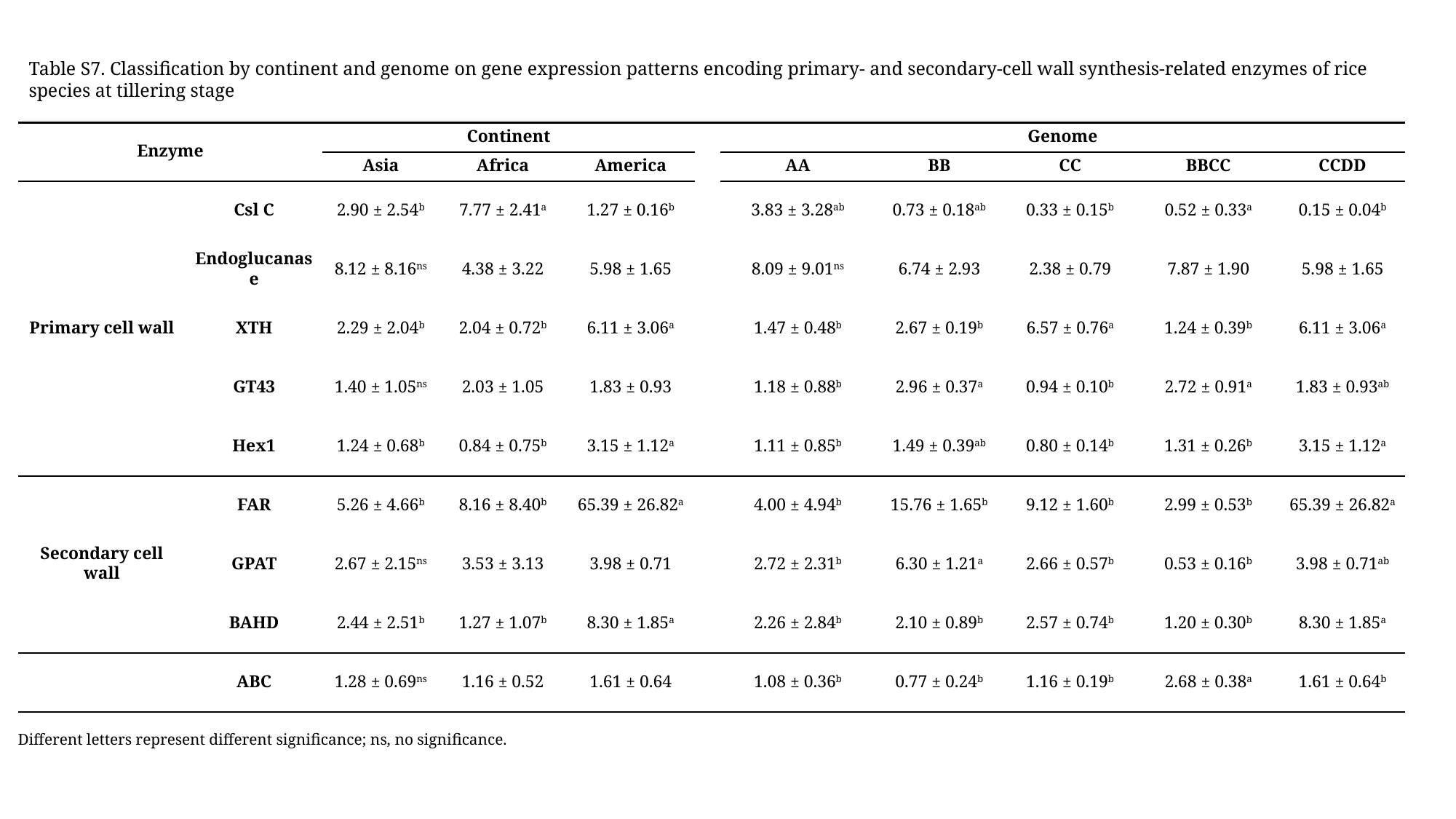

Table S7. Classification by continent and genome on gene expression patterns encoding primary- and secondary-cell wall synthesis-related enzymes of rice species at tillering stage
| Enzyme | | Continent | | | | Genome | | | | |
| --- | --- | --- | --- | --- | --- | --- | --- | --- | --- | --- |
| | | Asia | Africa | America | | AA | BB | CC | BBCC | CCDD |
| Primary cell wall | Csl C | 2.90 ± 2.54b | 7.77 ± 2.41a | 1.27 ± 0.16b | | 3.83 ± 3.28ab | 0.73 ± 0.18ab | 0.33 ± 0.15b | 0.52 ± 0.33a | 0.15 ± 0.04b |
| | Endoglucanase | 8.12 ± 8.16ns | 4.38 ± 3.22 | 5.98 ± 1.65 | | 8.09 ± 9.01ns | 6.74 ± 2.93 | 2.38 ± 0.79 | 7.87 ± 1.90 | 5.98 ± 1.65 |
| | XTH | 2.29 ± 2.04b | 2.04 ± 0.72b | 6.11 ± 3.06a | | 1.47 ± 0.48b | 2.67 ± 0.19b | 6.57 ± 0.76a | 1.24 ± 0.39b | 6.11 ± 3.06a |
| | GT43 | 1.40 ± 1.05ns | 2.03 ± 1.05 | 1.83 ± 0.93 | | 1.18 ± 0.88b | 2.96 ± 0.37a | 0.94 ± 0.10b | 2.72 ± 0.91a | 1.83 ± 0.93ab |
| | Hex1 | 1.24 ± 0.68b | 0.84 ± 0.75b | 3.15 ± 1.12a | | 1.11 ± 0.85b | 1.49 ± 0.39ab | 0.80 ± 0.14b | 1.31 ± 0.26b | 3.15 ± 1.12a |
| Secondary cell wall | FAR | 5.26 ± 4.66b | 8.16 ± 8.40b | 65.39 ± 26.82a | | 4.00 ± 4.94b | 15.76 ± 1.65b | 9.12 ± 1.60b | 2.99 ± 0.53b | 65.39 ± 26.82a |
| | GPAT | 2.67 ± 2.15ns | 3.53 ± 3.13 | 3.98 ± 0.71 | | 2.72 ± 2.31b | 6.30 ± 1.21a | 2.66 ± 0.57b | 0.53 ± 0.16b | 3.98 ± 0.71ab |
| | BAHD | 2.44 ± 2.51b | 1.27 ± 1.07b | 8.30 ± 1.85a | | 2.26 ± 2.84b | 2.10 ± 0.89b | 2.57 ± 0.74b | 1.20 ± 0.30b | 8.30 ± 1.85a |
| | ABC | 1.28 ± 0.69ns | 1.16 ± 0.52 | 1.61 ± 0.64 | | 1.08 ± 0.36b | 0.77 ± 0.24b | 1.16 ± 0.19b | 2.68 ± 0.38a | 1.61 ± 0.64b |
Different letters represent different significance; ns, no significance.

## Slide 9
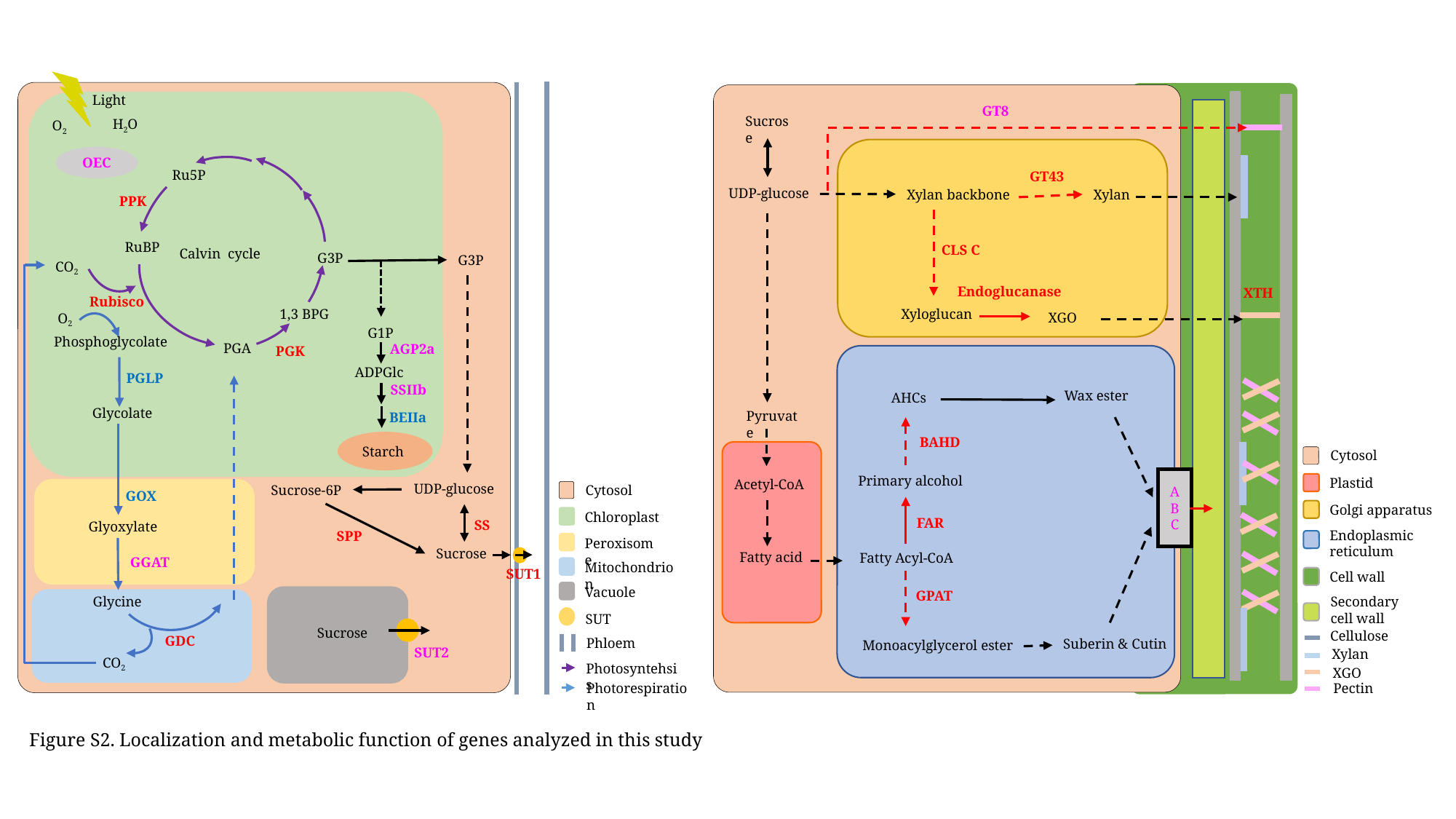

Light
H2O
O2
OEC
Ru5P
PPK
RuBP
Calvin cycle
G3P
G3P
CO2
Rubisco
1,3 BPG
O2
G1P
Phosphoglycolate
PGA
AGP2a
PGK
ADPGlc
PGLP
SSIIb
Glycolate
BEIIa
Starch
UDP-glucose
Cytosol
Chloroplast
Peroxisome
Mitochondrion
Vacuole
SUT
Phloem
Photosyntehsis
Photorespiration
Sucrose-6P
GOX
SS
Glyoxylate
SPP
Sucrose
GGAT
SUT1
Sucrose
SUT2
Glycine
GDC
CO2
GT8
Sucrose
GT43
UDP-glucose
Xylan backbone
Xylan
CLS C
Endoglucanase
XTH
Xyloglucan
XGO
Wax ester
AHCs
Primary alcohol
FAR
Fatty Acyl-CoA
GPAT
Monoacylglycerol ester
Pyruvate
BAHD
Cytosol
Plastid
A
B
C
Acetyl-CoA
Golgi apparatus
Endoplasmic reticulum
Fatty acid
Cell wall
Secondary cell wall
Cellulose
Suberin & Cutin
Xylan
XGO
Pectin
Figure S2. Localization and metabolic function of genes analyzed in this study

## Slide 10
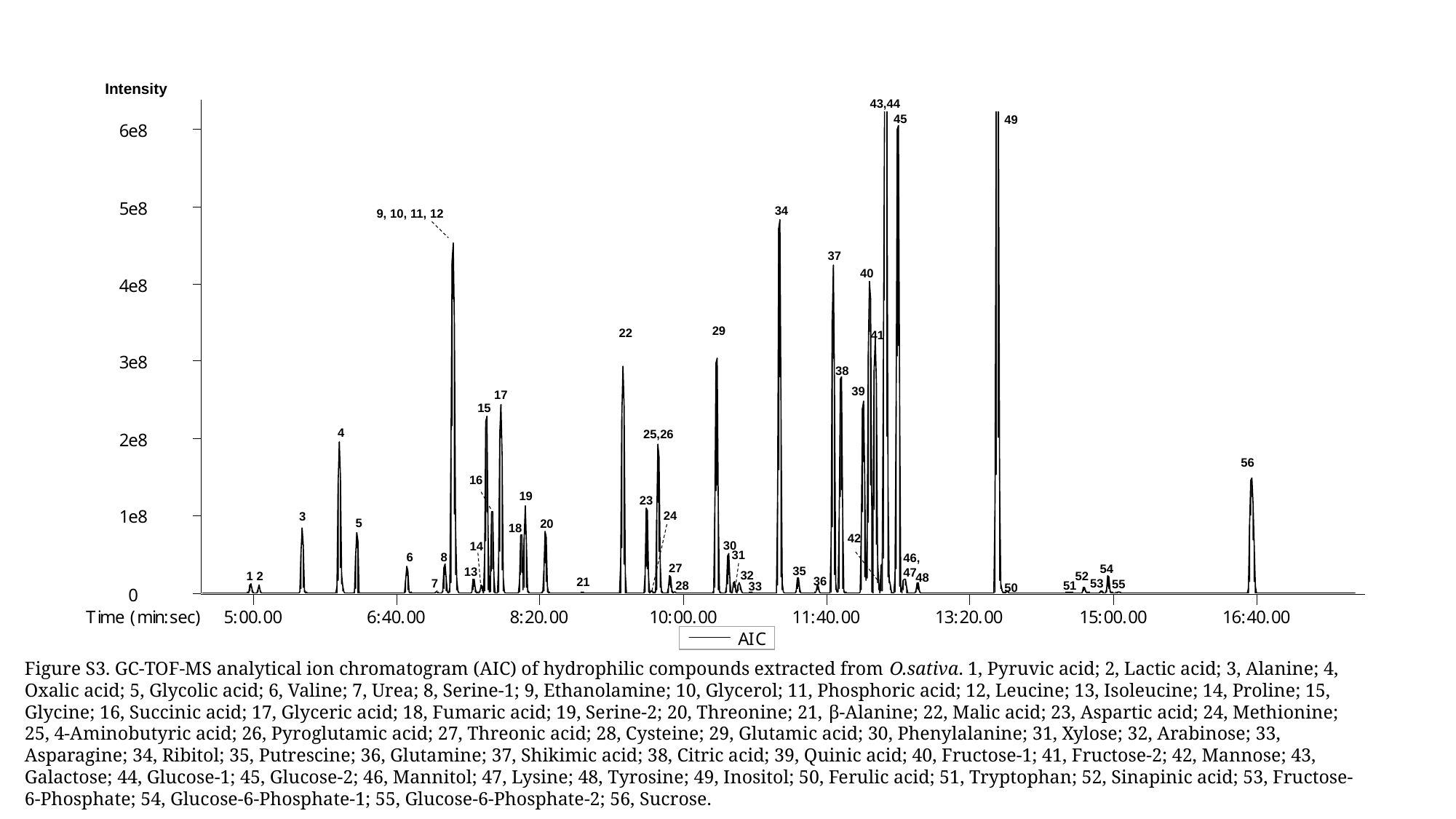

Intensity
43,44
45
49
34
9, 10, 11, 12
37
40
29
22
41
38
39
17
15
4
25,26
56
16
19
23
24
3
5
20
18
42
30
14
31
8
6
46,47
27
54
35
13
32
2
52
1
48
36
21
53
7
55
28
51
33
50
Figure S3. GC-TOF-MS analytical ion chromatogram (AIC) of hydrophilic compounds extracted from O.sativa. 1, Pyruvic acid; 2, Lactic acid; 3, Alanine; 4, Oxalic acid; 5, Glycolic acid; 6, Valine; 7, Urea; 8, Serine-1; 9, Ethanolamine; 10, Glycerol; 11, Phosphoric acid; 12, Leucine; 13, Isoleucine; 14, Proline; 15, Glycine; 16, Succinic acid; 17, Glyceric acid; 18, Fumaric acid; 19, Serine-2; 20, Threonine; 21, β-Alanine; 22, Malic acid; 23, Aspartic acid; 24, Methionine; 25, 4-Aminobutyric acid; 26, Pyroglutamic acid; 27, Threonic acid; 28, Cysteine; 29, Glutamic acid; 30, Phenylalanine; 31, Xylose; 32, Arabinose; 33, Asparagine; 34, Ribitol; 35, Putrescine; 36, Glutamine; 37, Shikimic acid; 38, Citric acid; 39, Quinic acid; 40, Fructose-1; 41, Fructose-2; 42, Mannose; 43, Galactose; 44, Glucose-1; 45, Glucose-2; 46, Mannitol; 47, Lysine; 48, Tyrosine; 49, Inositol; 50, Ferulic acid; 51, Tryptophan; 52, Sinapinic acid; 53, Fructose-6-Phosphate; 54, Glucose-6-Phosphate-1; 55, Glucose-6-Phosphate-2; 56, Sucrose.

## Slide 11
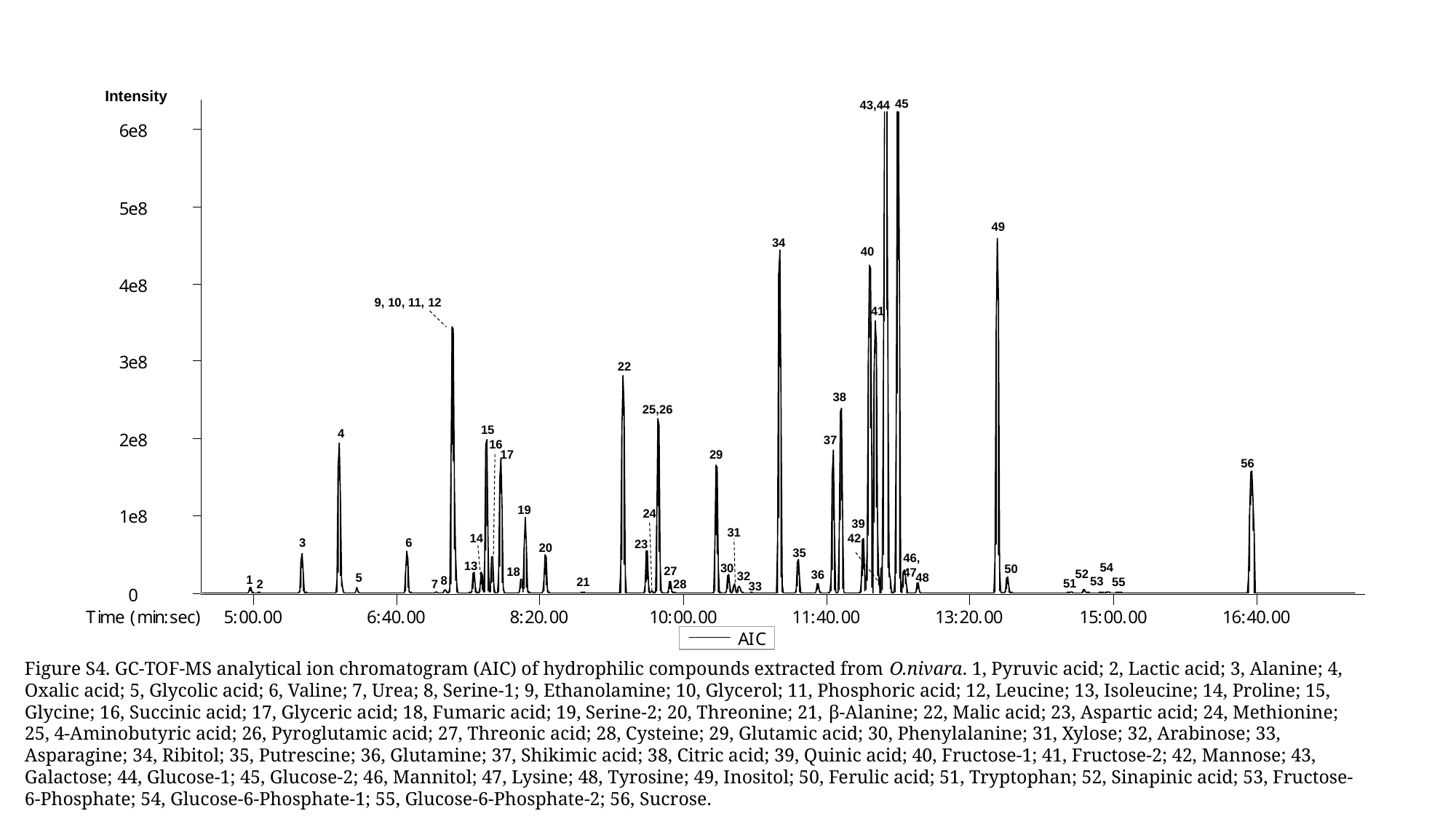

Intensity
45
43,44
49
34
40
9, 10, 11, 12
41
22
38
25,26
15
4
37
16
17
29
56
19
24
39
31
14
42
3
6
23
20
35
46,47
13
54
30
50
27
18
52
36
32
5
48
1
8
53
55
21
51
7
28
2
33
Figure S4. GC-TOF-MS analytical ion chromatogram (AIC) of hydrophilic compounds extracted from O.nivara. 1, Pyruvic acid; 2, Lactic acid; 3, Alanine; 4, Oxalic acid; 5, Glycolic acid; 6, Valine; 7, Urea; 8, Serine-1; 9, Ethanolamine; 10, Glycerol; 11, Phosphoric acid; 12, Leucine; 13, Isoleucine; 14, Proline; 15, Glycine; 16, Succinic acid; 17, Glyceric acid; 18, Fumaric acid; 19, Serine-2; 20, Threonine; 21, β-Alanine; 22, Malic acid; 23, Aspartic acid; 24, Methionine; 25, 4-Aminobutyric acid; 26, Pyroglutamic acid; 27, Threonic acid; 28, Cysteine; 29, Glutamic acid; 30, Phenylalanine; 31, Xylose; 32, Arabinose; 33, Asparagine; 34, Ribitol; 35, Putrescine; 36, Glutamine; 37, Shikimic acid; 38, Citric acid; 39, Quinic acid; 40, Fructose-1; 41, Fructose-2; 42, Mannose; 43, Galactose; 44, Glucose-1; 45, Glucose-2; 46, Mannitol; 47, Lysine; 48, Tyrosine; 49, Inositol; 50, Ferulic acid; 51, Tryptophan; 52, Sinapinic acid; 53, Fructose-6-Phosphate; 54, Glucose-6-Phosphate-1; 55, Glucose-6-Phosphate-2; 56, Sucrose.

## Slide 12
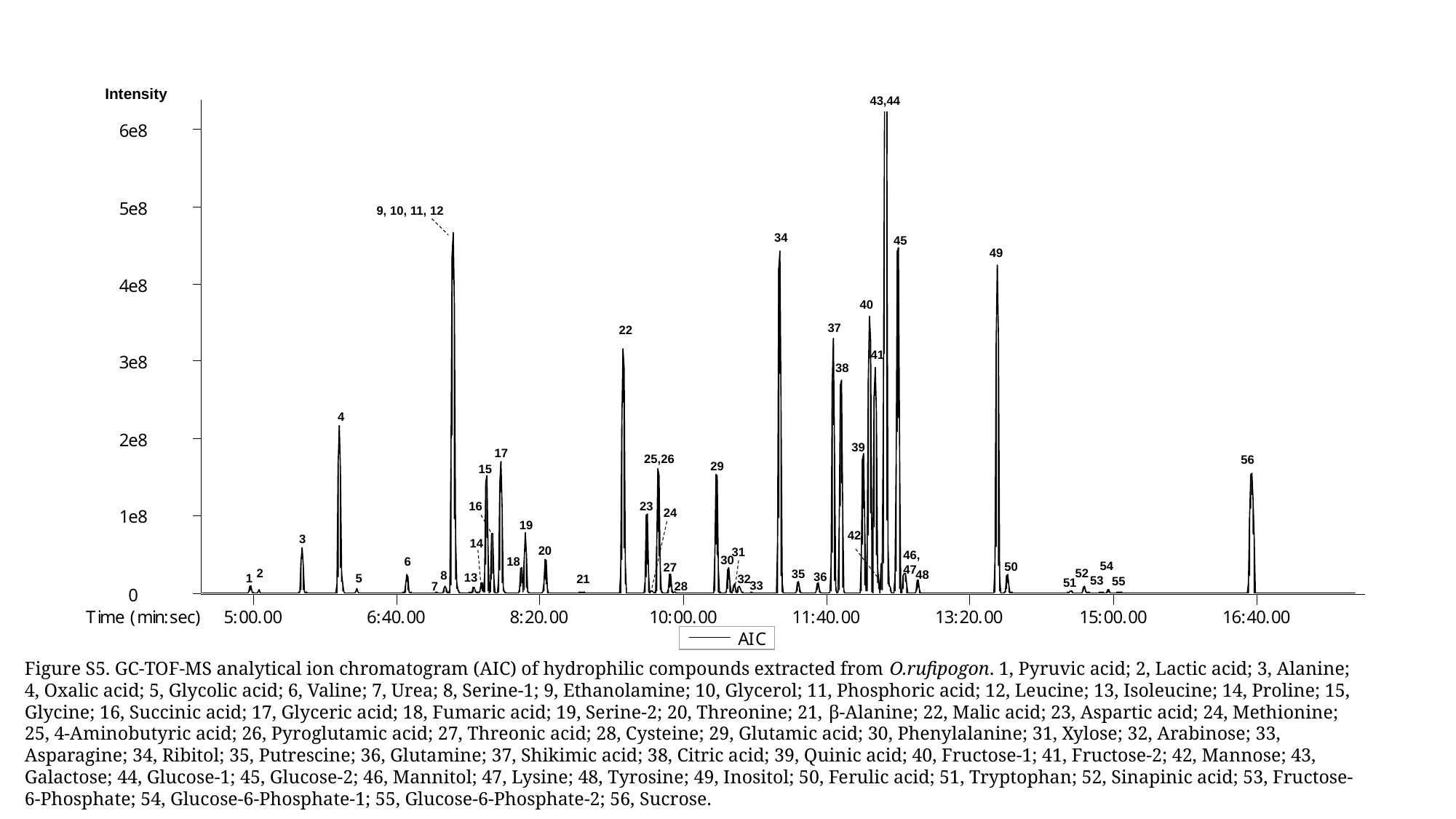

Intensity
43,44
9, 10, 11, 12
34
45
49
40
37
22
41
38
4
39
17
25,26
56
29
15
23
16
24
19
42
3
14
20
31
46,47
30
6
18
54
50
27
2
52
35
48
8
36
13
1
5
32
21
53
55
51
33
28
7
Figure S5. GC-TOF-MS analytical ion chromatogram (AIC) of hydrophilic compounds extracted from O.rufipogon. 1, Pyruvic acid; 2, Lactic acid; 3, Alanine; 4, Oxalic acid; 5, Glycolic acid; 6, Valine; 7, Urea; 8, Serine-1; 9, Ethanolamine; 10, Glycerol; 11, Phosphoric acid; 12, Leucine; 13, Isoleucine; 14, Proline; 15, Glycine; 16, Succinic acid; 17, Glyceric acid; 18, Fumaric acid; 19, Serine-2; 20, Threonine; 21, β-Alanine; 22, Malic acid; 23, Aspartic acid; 24, Methionine; 25, 4-Aminobutyric acid; 26, Pyroglutamic acid; 27, Threonic acid; 28, Cysteine; 29, Glutamic acid; 30, Phenylalanine; 31, Xylose; 32, Arabinose; 33, Asparagine; 34, Ribitol; 35, Putrescine; 36, Glutamine; 37, Shikimic acid; 38, Citric acid; 39, Quinic acid; 40, Fructose-1; 41, Fructose-2; 42, Mannose; 43, Galactose; 44, Glucose-1; 45, Glucose-2; 46, Mannitol; 47, Lysine; 48, Tyrosine; 49, Inositol; 50, Ferulic acid; 51, Tryptophan; 52, Sinapinic acid; 53, Fructose-6-Phosphate; 54, Glucose-6-Phosphate-1; 55, Glucose-6-Phosphate-2; 56, Sucrose.

## Slide 13
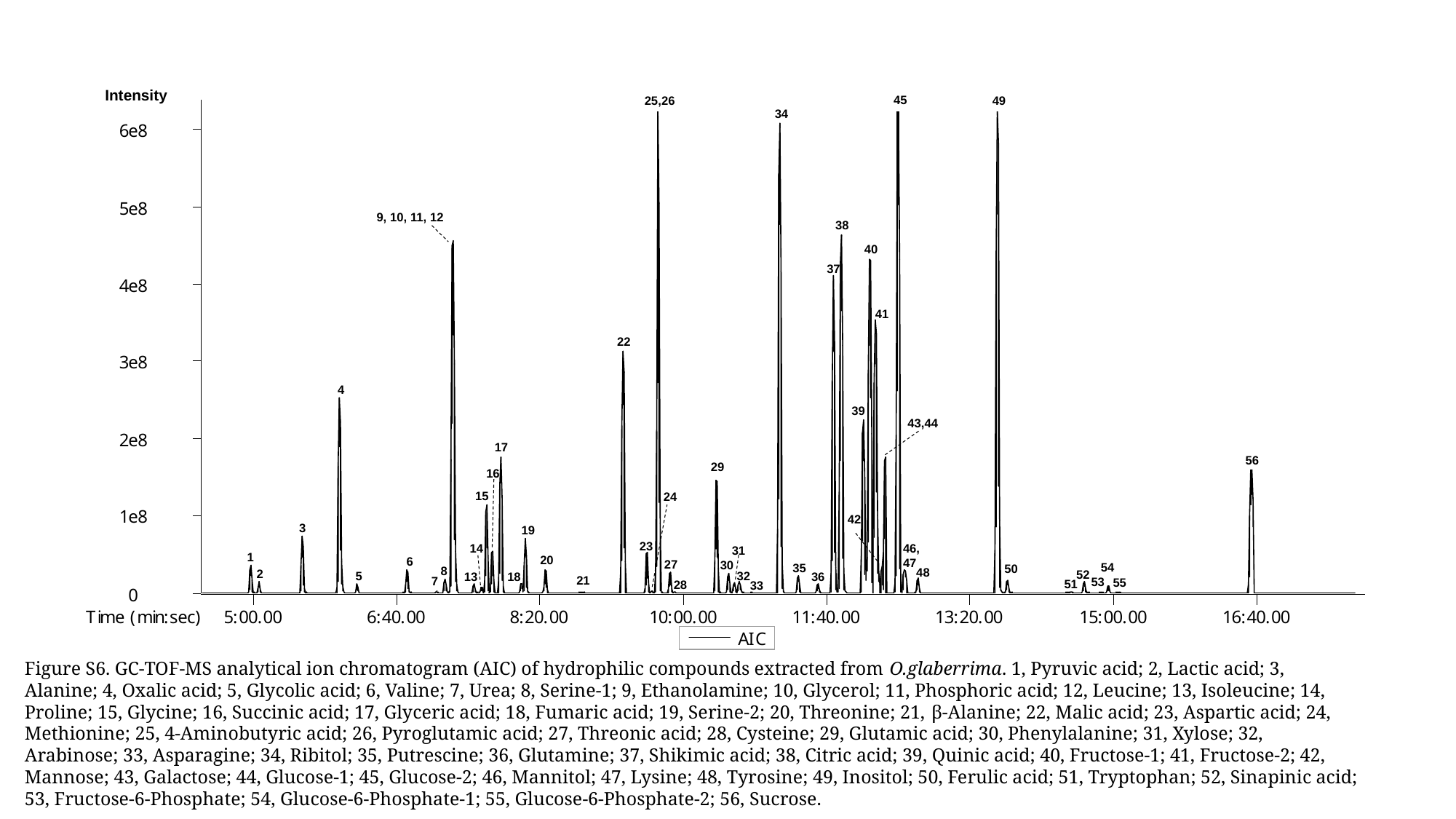

Intensity
45
25,26
49
34
9, 10, 11, 12
38
40
37
41
22
4
39
43,44
17
56
29
16
15
24
42
3
19
23
14
46,47
31
1
20
6
27
30
54
35
50
8
48
2
52
5
32
18
36
13
21
7
53
55
51
28
33
Figure S6. GC-TOF-MS analytical ion chromatogram (AIC) of hydrophilic compounds extracted from O.glaberrima. 1, Pyruvic acid; 2, Lactic acid; 3, Alanine; 4, Oxalic acid; 5, Glycolic acid; 6, Valine; 7, Urea; 8, Serine-1; 9, Ethanolamine; 10, Glycerol; 11, Phosphoric acid; 12, Leucine; 13, Isoleucine; 14, Proline; 15, Glycine; 16, Succinic acid; 17, Glyceric acid; 18, Fumaric acid; 19, Serine-2; 20, Threonine; 21, β-Alanine; 22, Malic acid; 23, Aspartic acid; 24, Methionine; 25, 4-Aminobutyric acid; 26, Pyroglutamic acid; 27, Threonic acid; 28, Cysteine; 29, Glutamic acid; 30, Phenylalanine; 31, Xylose; 32, Arabinose; 33, Asparagine; 34, Ribitol; 35, Putrescine; 36, Glutamine; 37, Shikimic acid; 38, Citric acid; 39, Quinic acid; 40, Fructose-1; 41, Fructose-2; 42, Mannose; 43, Galactose; 44, Glucose-1; 45, Glucose-2; 46, Mannitol; 47, Lysine; 48, Tyrosine; 49, Inositol; 50, Ferulic acid; 51, Tryptophan; 52, Sinapinic acid; 53, Fructose-6-Phosphate; 54, Glucose-6-Phosphate-1; 55, Glucose-6-Phosphate-2; 56, Sucrose.

## Slide 14
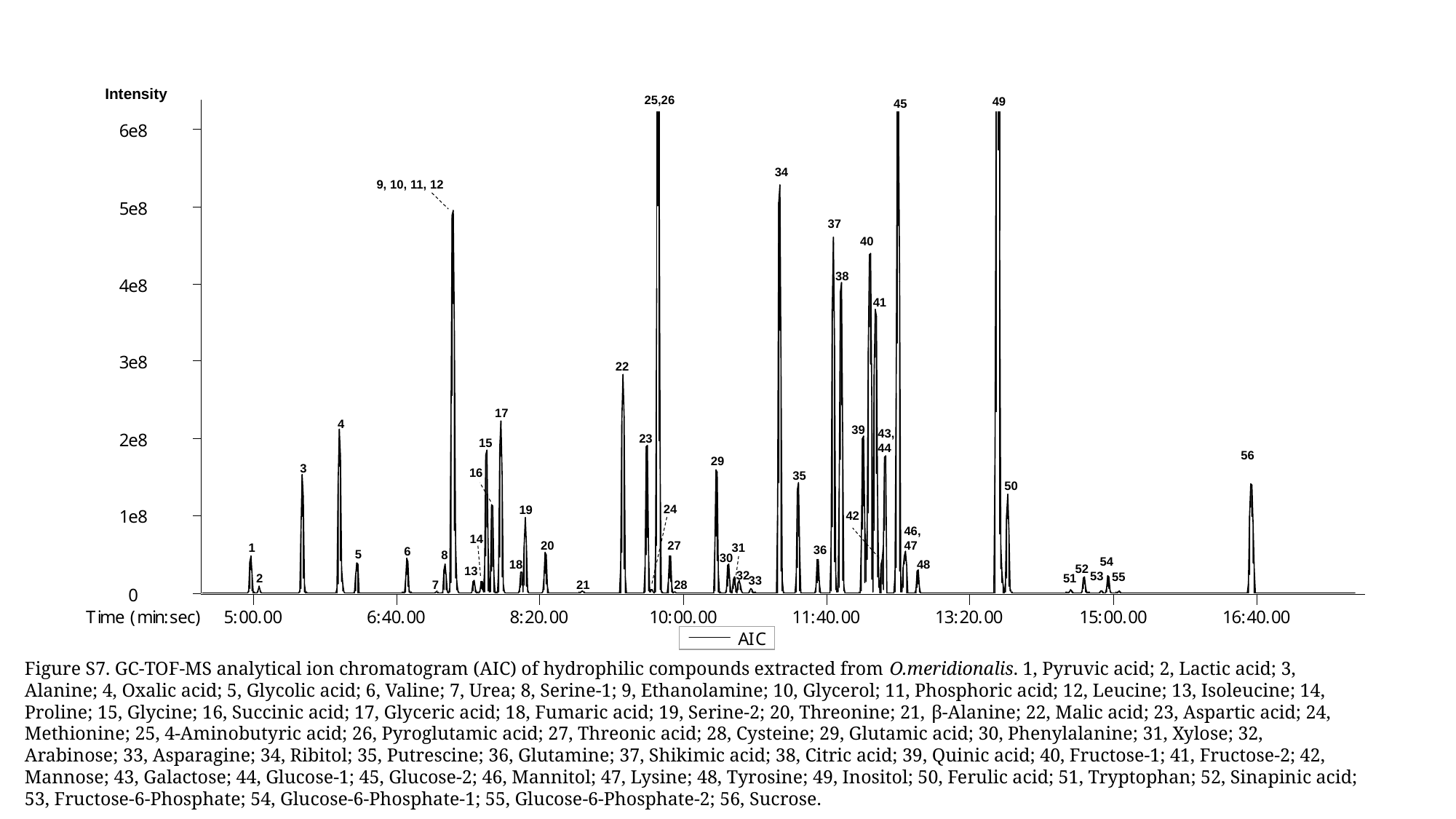

Intensity
25,26
49
45
34
9, 10, 11, 12
37
40
38
41
22
17
4
39
43,
44
23
15
56
29
3
16
35
50
24
19
42
46,47
14
27
20
31
1
36
6
5
8
30
54
18
48
52
13
32
53
55
51
2
33
21
7
28
Figure S7. GC-TOF-MS analytical ion chromatogram (AIC) of hydrophilic compounds extracted from O.meridionalis. 1, Pyruvic acid; 2, Lactic acid; 3, Alanine; 4, Oxalic acid; 5, Glycolic acid; 6, Valine; 7, Urea; 8, Serine-1; 9, Ethanolamine; 10, Glycerol; 11, Phosphoric acid; 12, Leucine; 13, Isoleucine; 14, Proline; 15, Glycine; 16, Succinic acid; 17, Glyceric acid; 18, Fumaric acid; 19, Serine-2; 20, Threonine; 21, β-Alanine; 22, Malic acid; 23, Aspartic acid; 24, Methionine; 25, 4-Aminobutyric acid; 26, Pyroglutamic acid; 27, Threonic acid; 28, Cysteine; 29, Glutamic acid; 30, Phenylalanine; 31, Xylose; 32, Arabinose; 33, Asparagine; 34, Ribitol; 35, Putrescine; 36, Glutamine; 37, Shikimic acid; 38, Citric acid; 39, Quinic acid; 40, Fructose-1; 41, Fructose-2; 42, Mannose; 43, Galactose; 44, Glucose-1; 45, Glucose-2; 46, Mannitol; 47, Lysine; 48, Tyrosine; 49, Inositol; 50, Ferulic acid; 51, Tryptophan; 52, Sinapinic acid; 53, Fructose-6-Phosphate; 54, Glucose-6-Phosphate-1; 55, Glucose-6-Phosphate-2; 56, Sucrose.

## Slide 15
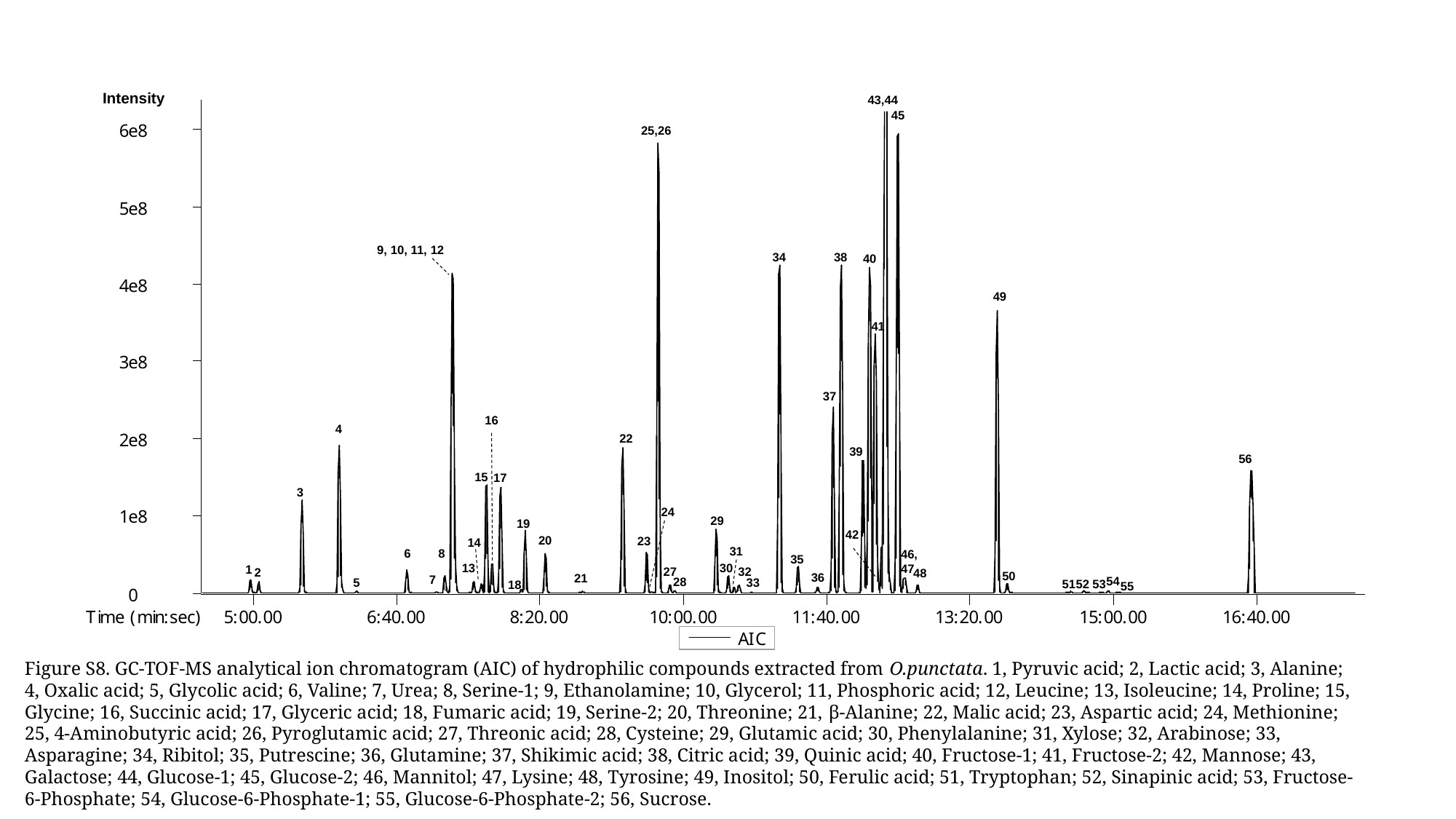

Intensity
43,44
45
25,26
9, 10, 11, 12
38
34
40
49
41
37
16
4
22
39
56
15
17
3
24
29
19
42
20
23
14
31
8
6
46,47
35
13
30
1
27
32
2
48
50
36
21
7
54
28
33
5
52
51
53
18
55
Figure S8. GC-TOF-MS analytical ion chromatogram (AIC) of hydrophilic compounds extracted from O.punctata. 1, Pyruvic acid; 2, Lactic acid; 3, Alanine; 4, Oxalic acid; 5, Glycolic acid; 6, Valine; 7, Urea; 8, Serine-1; 9, Ethanolamine; 10, Glycerol; 11, Phosphoric acid; 12, Leucine; 13, Isoleucine; 14, Proline; 15, Glycine; 16, Succinic acid; 17, Glyceric acid; 18, Fumaric acid; 19, Serine-2; 20, Threonine; 21, β-Alanine; 22, Malic acid; 23, Aspartic acid; 24, Methionine; 25, 4-Aminobutyric acid; 26, Pyroglutamic acid; 27, Threonic acid; 28, Cysteine; 29, Glutamic acid; 30, Phenylalanine; 31, Xylose; 32, Arabinose; 33, Asparagine; 34, Ribitol; 35, Putrescine; 36, Glutamine; 37, Shikimic acid; 38, Citric acid; 39, Quinic acid; 40, Fructose-1; 41, Fructose-2; 42, Mannose; 43, Galactose; 44, Glucose-1; 45, Glucose-2; 46, Mannitol; 47, Lysine; 48, Tyrosine; 49, Inositol; 50, Ferulic acid; 51, Tryptophan; 52, Sinapinic acid; 53, Fructose-6-Phosphate; 54, Glucose-6-Phosphate-1; 55, Glucose-6-Phosphate-2; 56, Sucrose.

## Slide 16
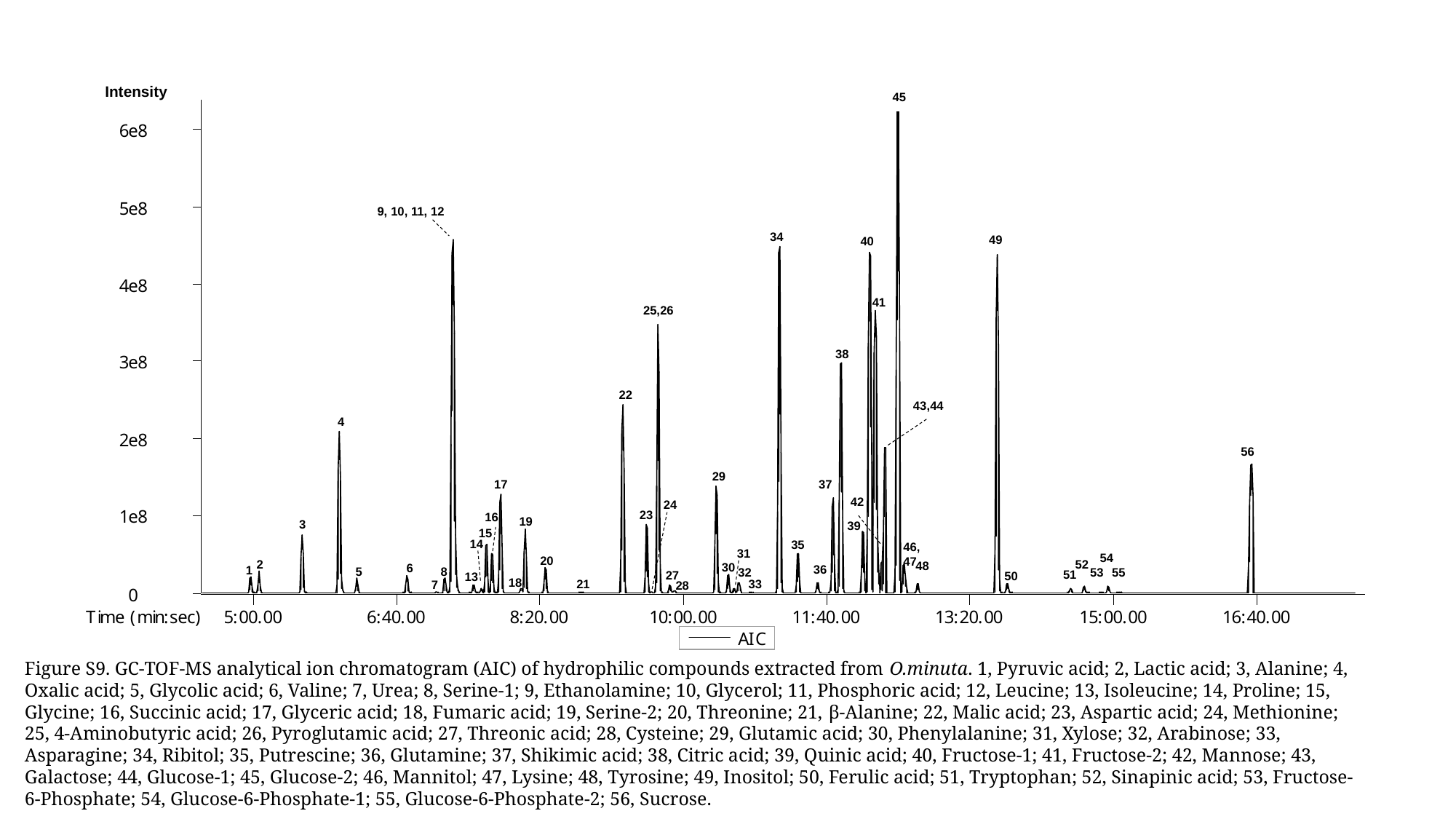

Intensity
45
9, 10, 11, 12
34
49
40
41
25,26
38
22
43,44
4
56
29
37
17
42
24
23
16
19
3
39
15
14
35
46,47
31
54
20
2
52
48
30
6
36
1
5
8
53
55
32
51
27
50
13
18
21
33
7
28
Figure S9. GC-TOF-MS analytical ion chromatogram (AIC) of hydrophilic compounds extracted from O.minuta. 1, Pyruvic acid; 2, Lactic acid; 3, Alanine; 4, Oxalic acid; 5, Glycolic acid; 6, Valine; 7, Urea; 8, Serine-1; 9, Ethanolamine; 10, Glycerol; 11, Phosphoric acid; 12, Leucine; 13, Isoleucine; 14, Proline; 15, Glycine; 16, Succinic acid; 17, Glyceric acid; 18, Fumaric acid; 19, Serine-2; 20, Threonine; 21, β-Alanine; 22, Malic acid; 23, Aspartic acid; 24, Methionine; 25, 4-Aminobutyric acid; 26, Pyroglutamic acid; 27, Threonic acid; 28, Cysteine; 29, Glutamic acid; 30, Phenylalanine; 31, Xylose; 32, Arabinose; 33, Asparagine; 34, Ribitol; 35, Putrescine; 36, Glutamine; 37, Shikimic acid; 38, Citric acid; 39, Quinic acid; 40, Fructose-1; 41, Fructose-2; 42, Mannose; 43, Galactose; 44, Glucose-1; 45, Glucose-2; 46, Mannitol; 47, Lysine; 48, Tyrosine; 49, Inositol; 50, Ferulic acid; 51, Tryptophan; 52, Sinapinic acid; 53, Fructose-6-Phosphate; 54, Glucose-6-Phosphate-1; 55, Glucose-6-Phosphate-2; 56, Sucrose.

## Slide 17
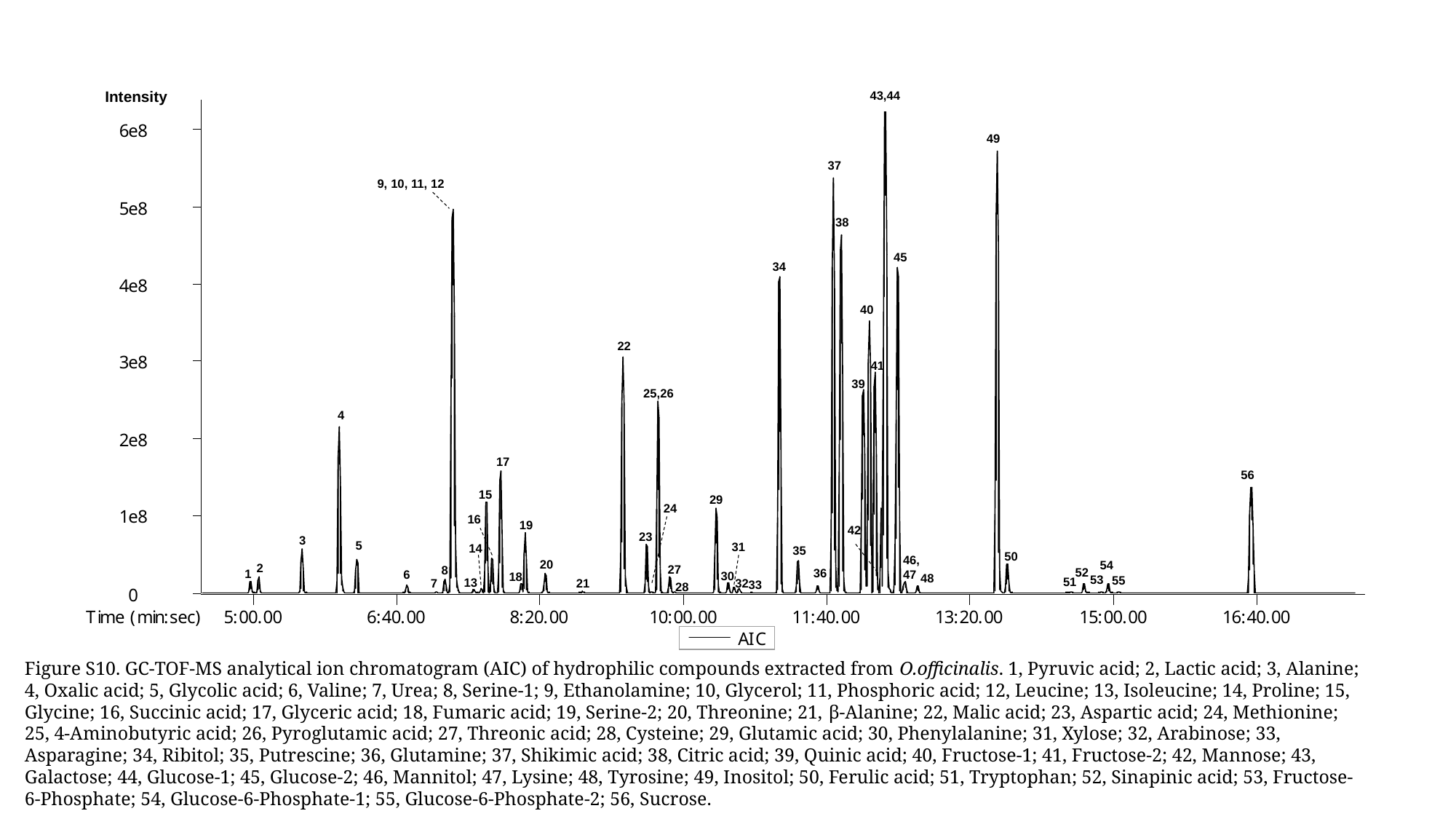

Intensity
43,44
49
37
9, 10, 11, 12
38
45
34
40
22
41
39
25,26
4
17
56
15
29
24
16
19
42
23
3
5
31
14
35
50
46,47
20
54
2
27
8
52
36
1
6
30
18
48
53
55
51
13
32
21
7
33
28
Figure S10. GC-TOF-MS analytical ion chromatogram (AIC) of hydrophilic compounds extracted from O.officinalis. 1, Pyruvic acid; 2, Lactic acid; 3, Alanine; 4, Oxalic acid; 5, Glycolic acid; 6, Valine; 7, Urea; 8, Serine-1; 9, Ethanolamine; 10, Glycerol; 11, Phosphoric acid; 12, Leucine; 13, Isoleucine; 14, Proline; 15, Glycine; 16, Succinic acid; 17, Glyceric acid; 18, Fumaric acid; 19, Serine-2; 20, Threonine; 21, β-Alanine; 22, Malic acid; 23, Aspartic acid; 24, Methionine; 25, 4-Aminobutyric acid; 26, Pyroglutamic acid; 27, Threonic acid; 28, Cysteine; 29, Glutamic acid; 30, Phenylalanine; 31, Xylose; 32, Arabinose; 33, Asparagine; 34, Ribitol; 35, Putrescine; 36, Glutamine; 37, Shikimic acid; 38, Citric acid; 39, Quinic acid; 40, Fructose-1; 41, Fructose-2; 42, Mannose; 43, Galactose; 44, Glucose-1; 45, Glucose-2; 46, Mannitol; 47, Lysine; 48, Tyrosine; 49, Inositol; 50, Ferulic acid; 51, Tryptophan; 52, Sinapinic acid; 53, Fructose-6-Phosphate; 54, Glucose-6-Phosphate-1; 55, Glucose-6-Phosphate-2; 56, Sucrose.

## Slide 18
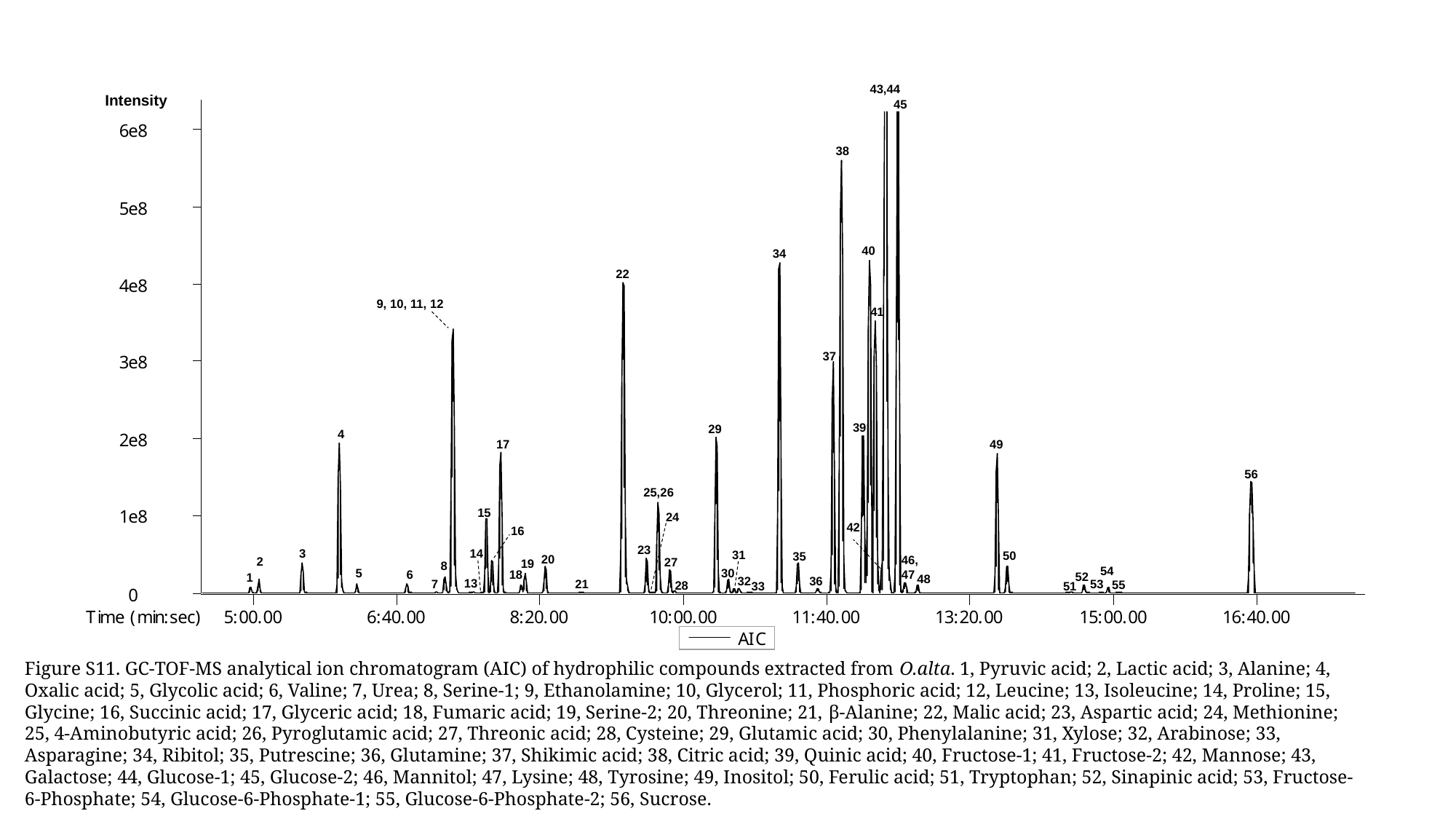

43,44
Intensity
45
38
40
34
22
9, 10, 11, 12
41
37
39
29
4
17
49
56
25,26
15
24
42
16
23
3
14
31
50
35
20
46,47
2
27
19
8
54
5
30
6
18
52
1
48
32
36
13
7
21
53
55
28
33
51
Figure S11. GC-TOF-MS analytical ion chromatogram (AIC) of hydrophilic compounds extracted from O.alta. 1, Pyruvic acid; 2, Lactic acid; 3, Alanine; 4, Oxalic acid; 5, Glycolic acid; 6, Valine; 7, Urea; 8, Serine-1; 9, Ethanolamine; 10, Glycerol; 11, Phosphoric acid; 12, Leucine; 13, Isoleucine; 14, Proline; 15, Glycine; 16, Succinic acid; 17, Glyceric acid; 18, Fumaric acid; 19, Serine-2; 20, Threonine; 21, β-Alanine; 22, Malic acid; 23, Aspartic acid; 24, Methionine; 25, 4-Aminobutyric acid; 26, Pyroglutamic acid; 27, Threonic acid; 28, Cysteine; 29, Glutamic acid; 30, Phenylalanine; 31, Xylose; 32, Arabinose; 33, Asparagine; 34, Ribitol; 35, Putrescine; 36, Glutamine; 37, Shikimic acid; 38, Citric acid; 39, Quinic acid; 40, Fructose-1; 41, Fructose-2; 42, Mannose; 43, Galactose; 44, Glucose-1; 45, Glucose-2; 46, Mannitol; 47, Lysine; 48, Tyrosine; 49, Inositol; 50, Ferulic acid; 51, Tryptophan; 52, Sinapinic acid; 53, Fructose-6-Phosphate; 54, Glucose-6-Phosphate-1; 55, Glucose-6-Phosphate-2; 56, Sucrose.

## Slide 19
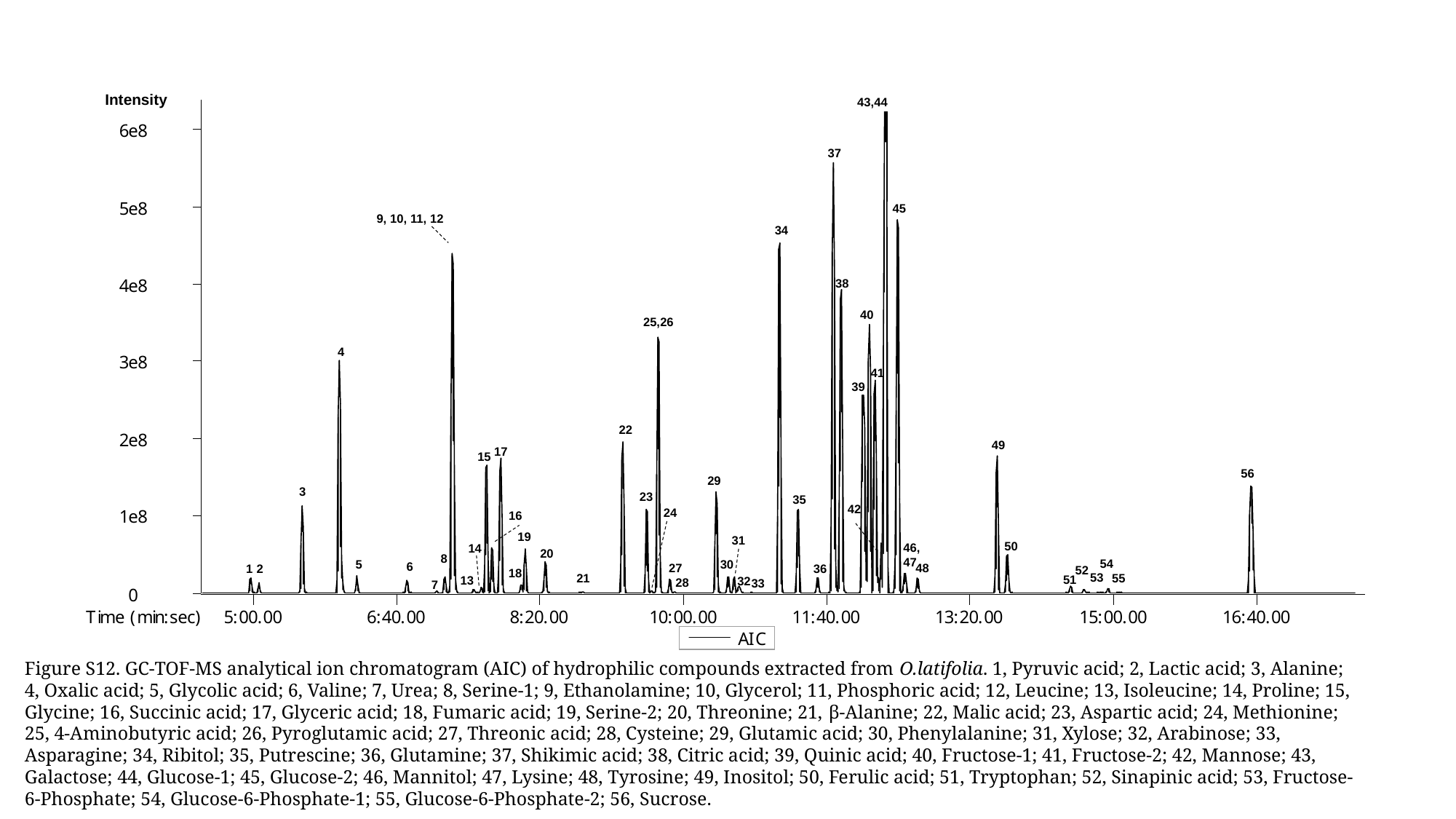

Intensity
43,44
37
45
9, 10, 11, 12
34
38
40
25,26
4
41
39
22
49
17
15
56
29
3
23
35
42
24
16
19
31
50
46,47
14
20
8
54
5
30
6
27
48
1
36
2
52
18
53
21
55
51
13
32
28
33
7
Figure S12. GC-TOF-MS analytical ion chromatogram (AIC) of hydrophilic compounds extracted from O.latifolia. 1, Pyruvic acid; 2, Lactic acid; 3, Alanine; 4, Oxalic acid; 5, Glycolic acid; 6, Valine; 7, Urea; 8, Serine-1; 9, Ethanolamine; 10, Glycerol; 11, Phosphoric acid; 12, Leucine; 13, Isoleucine; 14, Proline; 15, Glycine; 16, Succinic acid; 17, Glyceric acid; 18, Fumaric acid; 19, Serine-2; 20, Threonine; 21, β-Alanine; 22, Malic acid; 23, Aspartic acid; 24, Methionine; 25, 4-Aminobutyric acid; 26, Pyroglutamic acid; 27, Threonic acid; 28, Cysteine; 29, Glutamic acid; 30, Phenylalanine; 31, Xylose; 32, Arabinose; 33, Asparagine; 34, Ribitol; 35, Putrescine; 36, Glutamine; 37, Shikimic acid; 38, Citric acid; 39, Quinic acid; 40, Fructose-1; 41, Fructose-2; 42, Mannose; 43, Galactose; 44, Glucose-1; 45, Glucose-2; 46, Mannitol; 47, Lysine; 48, Tyrosine; 49, Inositol; 50, Ferulic acid; 51, Tryptophan; 52, Sinapinic acid; 53, Fructose-6-Phosphate; 54, Glucose-6-Phosphate-1; 55, Glucose-6-Phosphate-2; 56, Sucrose.

## Slide 20
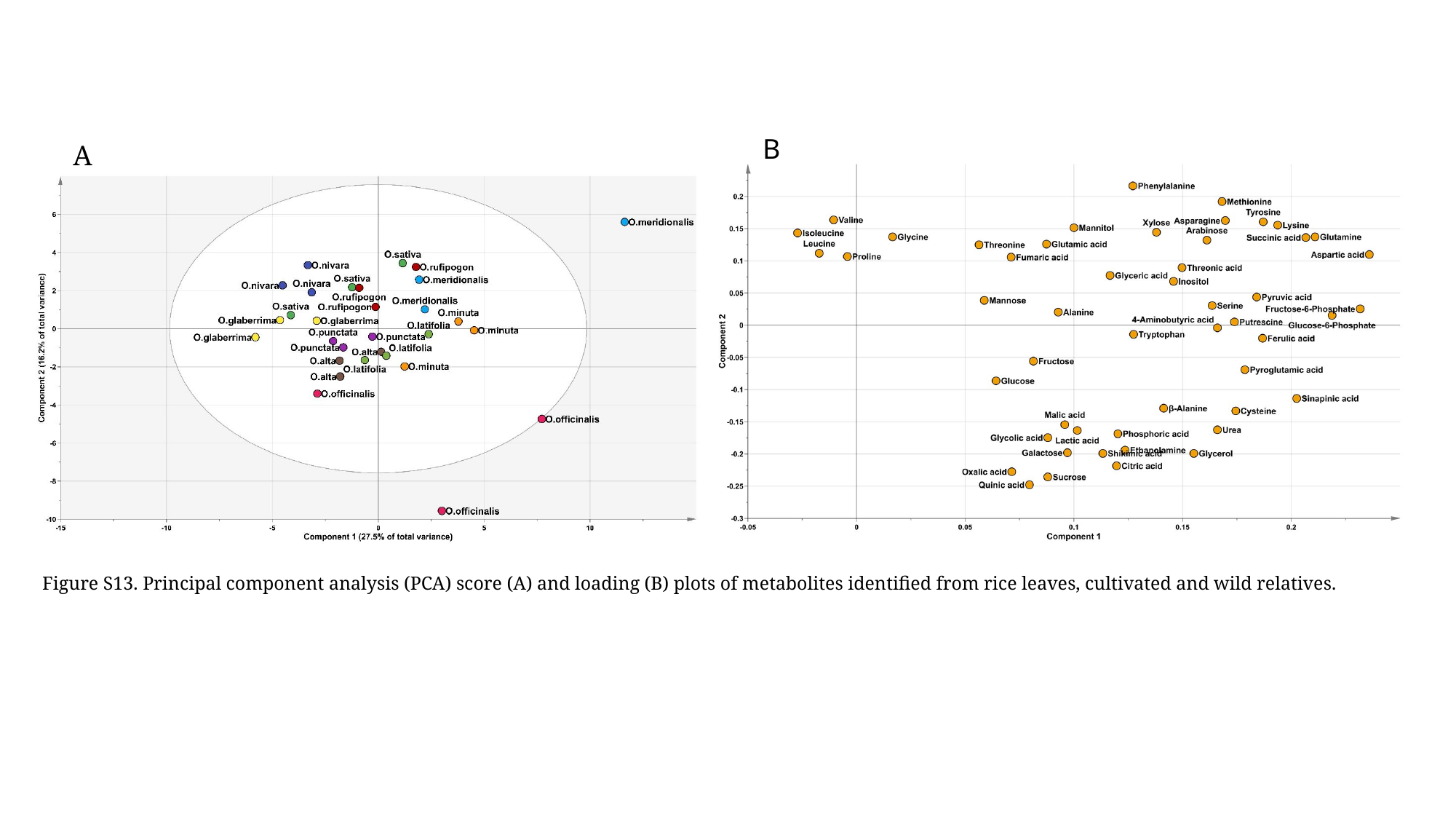

B
A
Figure S13. Principal component analysis (PCA) score (A) and loading (B) plots of metabolites identified from rice leaves, cultivated and wild relatives.

## Slide 21
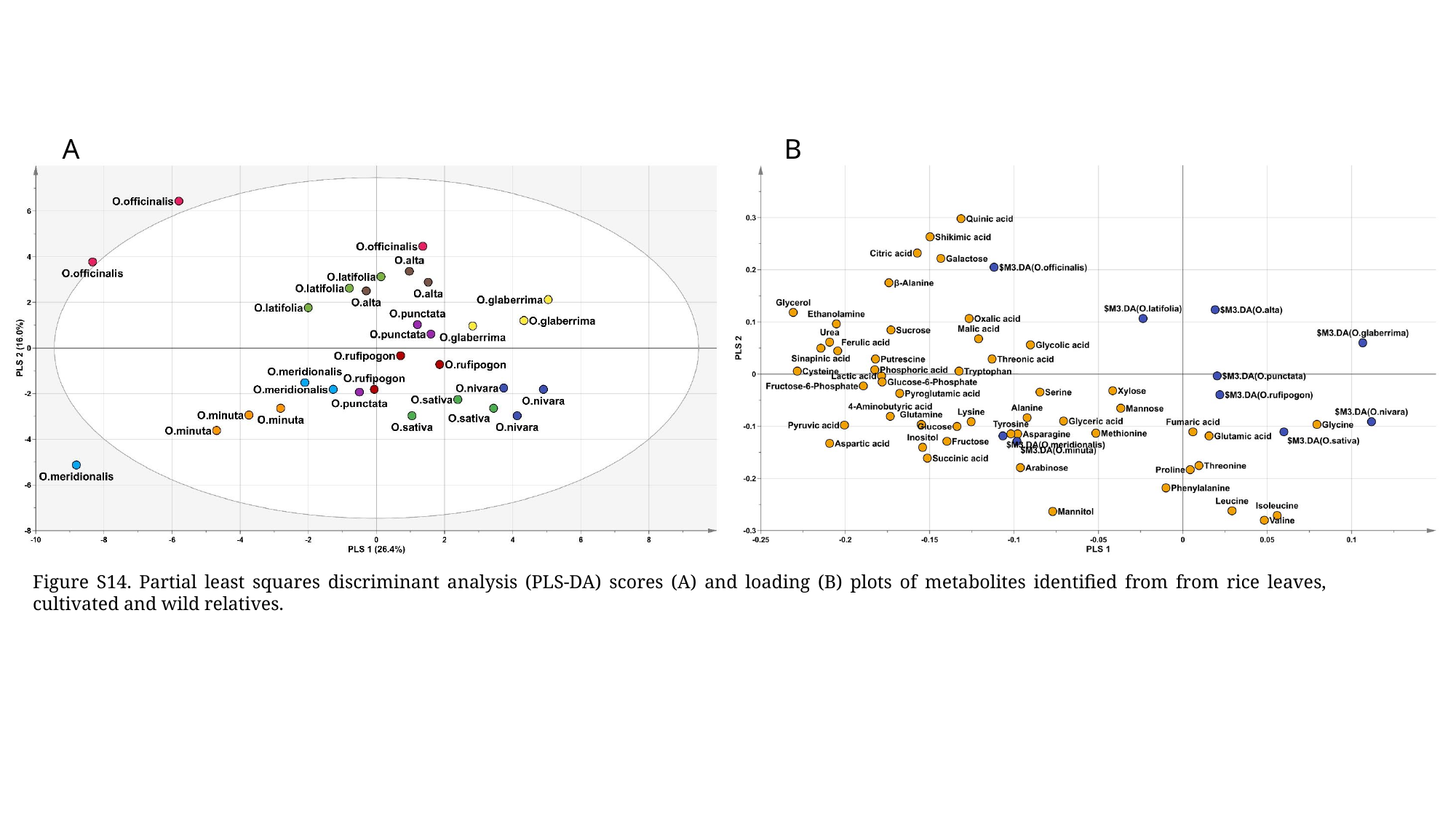

A
B
Figure S14. Partial least squares discriminant analysis (PLS-DA) scores (A) and loading (B) plots of metabolites identified from from rice leaves, cultivated and wild relatives.

## Slide 22
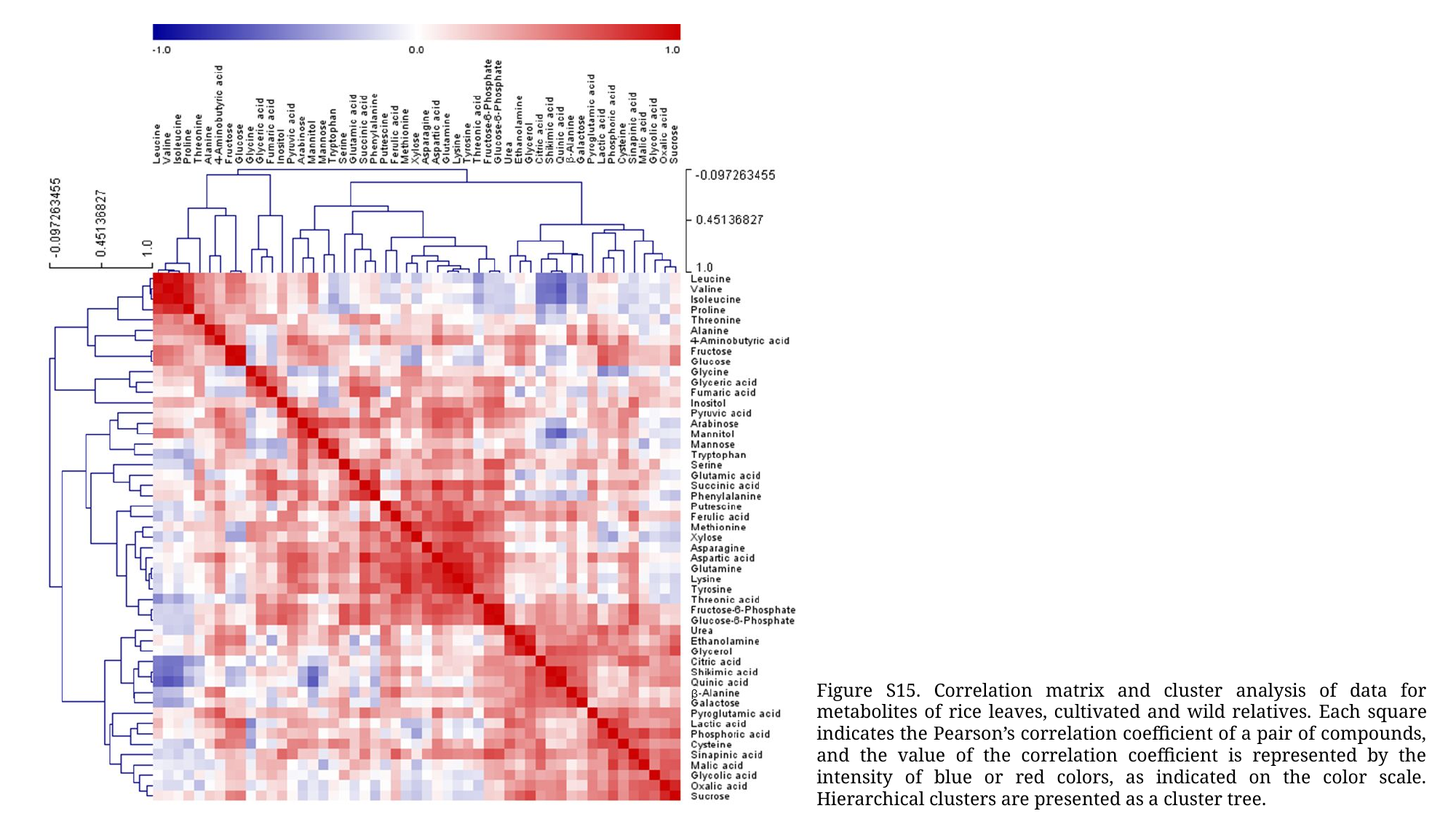

Figure S15. Correlation matrix and cluster analysis of data for metabolites of rice leaves, cultivated and wild relatives. Each square indicates the Pearson’s correlation coefficient of a pair of compounds, and the value of the correlation coefficient is represented by the intensity of blue or red colors, as indicated on the color scale. Hierarchical clusters are presented as a cluster tree.
